# An *ex vivo* model of *Toxoplasma* recrudescence reveals developmental plasticity of the bradyzoite stage

**DOI:** 10.1128/mbio.01836-23

**Published:** 2023-09-07

**Authors:** Edward A. Vizcarra, Amber L. Goerner, Arzu Ulu, David D. Hong, Kristina V. Bergersen, Michael A. Talavera, Karine Le Roch, Emma H. Wilson, Michael W. White

**Affiliations:** 1 Division of Biomedical Sciences, School of Medicine, University of California, Riverside, California, USA; 2 Division of Infectious Disease and International Medicine, Department of Internal Medicine, Morsani College of Medicine, University of South Florida, Tampa, Florida, USA; 3 Department of Molecular, Cell, and Systems Biology, University of California, Riverside, California, USA; Stanford University, Stanford, California, USA; University of California Davis, Davis, California, USA

**Keywords:** Apicomplexa, *Toxoplasma gondii*, development, tissue cyst, latency, chronic toxoplasmosis

## Abstract

The recrudescence of *Toxoplasma* cysts is the cause of clinical disease in the immunocompromised. Although *Toxoplasma* has been a useful parasite model for decades because it is relatively easy to genetically modify and culture, attempts to generate and study the recrudescence of tissue cysts have come up short with cell culture-adapted strains generating low numbers of tissue cysts *in vivo*. Taking advantage of a new *ex vivo* model of *Toxoplasma* recrudescence that uses a Type II ME49 strain unadapted to cell culture, we determined the cell biology, gene expression, and host cell dependency that define bradyzoite-cyst reactivation. Bradyzoite infection of fibroblasts and astrocytes produced sequential tachyzoite growth stages with pre-programmed kinetics; thus, an initial fast-growing stage was followed by a slow-growing replicating form. *In vivo* infections demonstrated that only fast growth tachyzoites, and not parasites post-growth shift, led to successful parasite dissemination to the brain and peripheral organs. In astrocytes, cells that reside in the central nervous system (CNS), bradyzoites initiated an additional recrudescent pathway involving brady-brady replication, which is a pathway not observed in fibroblasts. To investigate the molecular basis of growth and cell-dependent reactivation pathways, single-cell mRNA sequencing was performed on recrudescing parasites, revealing distinct gene signatures of these parasite populations and confirming multifunctionality of the original *ex vivo* bradyzoite population. This revised model of *Toxoplasma* recrudescence uncovers previously unknown complexity in the clinically important bradyzoite stage of the parasite, which opens the door to further study these novel developmental features of the *Toxoplasma* intermediate life cycle.

**IMPORTANCE:**

The classical depiction of the *Toxoplasma* lifecycle is bradyzoite excystation conversion to tachyzoites, cell lysis, and immune control, followed by the reestablishment of bradyzoites and cysts. In contrast, we show that tachyzoite growth slows independent of the host immune response at a predictable time point following excystation. Furthermore, we demonstrate a host cell-dependent pathway of continuous amplification of the cyst-forming bradyzoite population. The developmental plasticity of the excysted bradyzoites further underlines the critical role the cyst plays in the flexibility of the lifecycle of this ubiquitous parasite. This revised model of *Toxoplasma* recrudescence uncovers previously unknown complexity in the clinically important bradyzoite stage of the parasite, which opens the door to further study these novel developmental features of the *Toxoplasma* intermediate life cycle.

## INTRODUCTION

The reactivation of the *Toxoplasma* tissue cyst is a significant health threat to people who are chronically infected and is life-threatening to infected individuals who are or become immunocompromised. Individuals acquire their initial *Toxoplasma* infection by ingesting oocysts from environmental sources or tissue cysts from contaminated food products. Oocysts and tissue cysts are exposed to proteases in the GI tract that release the sporozoite and bradyzoite stages, respectively, to invade the epithelial cells lining the gut. The time from localized infection to systemic escape is relatively short in mice. After 2 h post-oral oocyst infection, *Toxoplasma* parasites have infected the intestinal epithelium and by 8 h the infection can be detected in the mesenteric lymph nodes (1). After 6–7 days, the tissue cyst form can be detected in the brain of the mice orally infected with the oocyst stage (2). The short timeframe from initial infection to the establishment of chronic disease makes it difficult to prevent *Toxoplasma* infection, which accounts for the widespread distribution of this parasite. It is estimated that one-third of human populations are infected with this pathogen (3). Healthy individuals are at low risk for clinical complications from toxoplasmosis; however, those with compromised immune systems are susceptible to severe clinical disease (4). *Toxoplasma* is one of the most frequent causes of inflammation of the uvea layer of the eye (3), and in some cases these infections result in the loss of vision. Relapsing disease is common in the first 2 years following a primary lesion diagnosis, especially in elderly patients (5). Prior to the introduction of highly active antiretroviral therapy (HAART), *Toxoplasma* caused frequent life-threatening encephalitis in AIDS patients, with reactivating disease thought to be the most common cause (3). Today, *Toxoplasma* infections in AIDS patients remain a significant clinical threat to large populations that have poor access to HIV therapies, and this will remain until there is an HIV cure and/or a therapy to eliminate the chronic tissue cyst stage of *Toxoplasma* development.

Recrudescence of the *Toxoplasma* bradyzoite tissue cyst is central to the reactivation of toxoplasmosis. However, despite the importance of this developmental process, the developmental biology of the bradyzoite is poorly understood. Studies of severely immunosuppressed mice indicate that *Toxoplasma* reactivation preferentially occurs in the brain’s frontal and parietal cortex grey matter (6). Attempts to define the stages of cyst parasite recrudescence in immunosuppressed mouse models and in post-mortem examinations of AIDS patients have observed both bradyzoites and tachyzoites in foci of reactivation (7, 8). Consequently, the original cyst rupture, the sequence of parasite developmental steps, and host cell contributions have not been discerned (7, 8). Because of the challenges in studying *Toxoplasma* reactivation in animal models, it is critical to develop an *in vitro* model of bradyzoite reactivation that could accelerate efforts to understand these processes at the cell and molecular level.

In this paper, we describe a new *ex vivo* model of *Toxoplasma* recrudescence using a robust cyst-forming strain that has never been adapted to cell culture. Our results reveal that instead of a linear bradyzoite-to-tachyzoite event, there are complex pathways of development during bradyzoite recrudescence, which are both host cell independent and host cell dependent.

## RESULTS

### Adaptation to cell culture leads to the loss of *Toxoplasma* developmental competency

**Fig 1:**
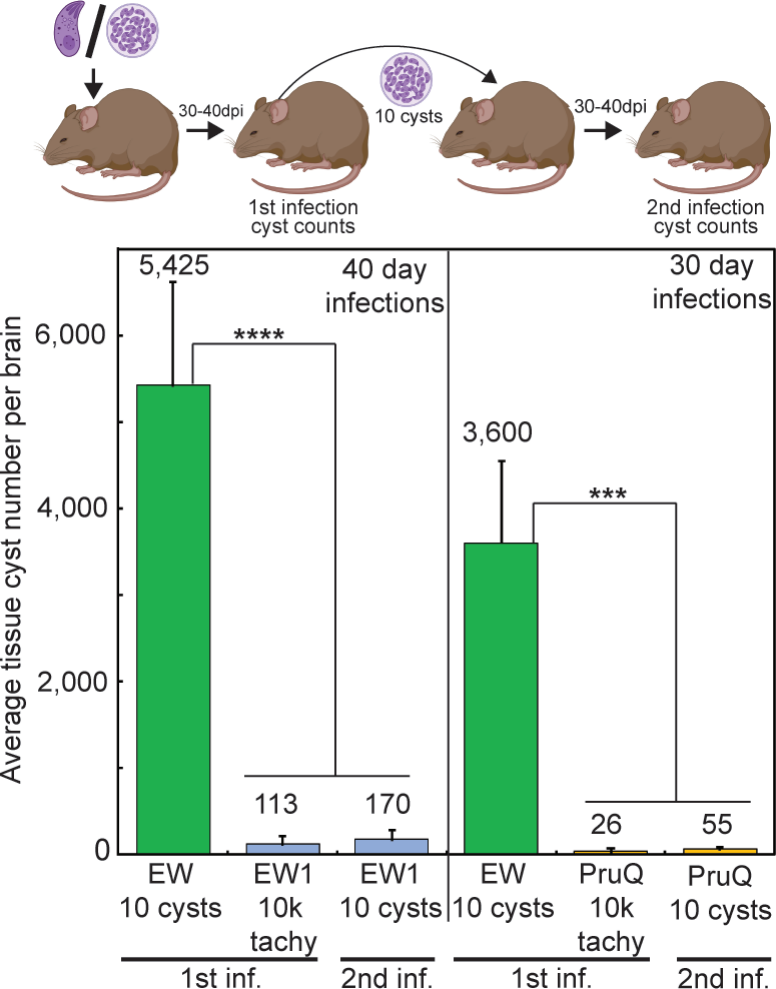
Adaption of ME49EW parasites to grow in fibroblasts leads to permanent loss of cyst development in mice. CBA/J mouse infections (five mice per group) are detailed in the diagram above the graph. Mice were infected with unadapted ME49EW tissue cysts (10 cysts i.p.) followed by brain cyst counts at 30 or 40 d.p.i. Tachyzoites (10^4^, i.p.) from HFF cultures infected with HFF-adapted strains, ME49EW1 or PruQ, were used for primary infections in mice with cyst counts determined at 30 d.p.i. (PruQ) or 40 d.p.i. (EW1). Secondary infections with ME49EW1 or PruQ tissue cysts (10 cysts, i.p.) from the matching primary infected mice were performed with brain cyst counts determined at 40 and 30 d.p.i., respectively. Methods for quantifying tissue cysts in brain homogenates were previously described (10). Average brain cyst numbers are indicated above each graph bar. Statistically significant differences in the brain cyst counts of native ME49EW bradyzoite vs. HFF-adapted strain infections are indicated (****P < 0.0001 for 40 d.p.i. mice and ***P < 0.001 for 30 d.p.i. mice).

One of the major challenges in studying the bradyzoite-tissue cyst stage of *Toxoplasma* has been the preservation of developmental competency in experimental models, especially cell culture systems. Type II strains have been regularly studied as they represent the most common genotype associated with human infections (9). The Type II ME49 strain used here (designated ME49EW) has been exclusively maintained *in vivo* by alternating passage through resistant and sensitive mouse strains in order to establish a stable chronic infection. ME49EW infections reliably lead to high numbers of cysts in murine brain tissue (10). From an intraperitoneal (i.p.) injection of 10 cysts, the ME49EW strain consistently produced thousands of tissue cysts (first infection, Fig. 1). To demonstrate the problems associated with cell culture adaptation, we infected human foreskin fibroblasts (HFF) with ME49EW bradyzoites. After six attempts (~30 million total bradyzoites plated), an HFF-adapted parasite line spontaneously emerged and was cloned (designated ME49EW1 strain). An infection of 10,000 ME49EW1 tachyzoites was well tolerated by a sensitive mouse strain (CBA/j); however, the competency to produce tissue cysts in brain tissue was dramatically reduced (Fig. 1, EW1-1st inf.). The 48-fold reduction in ME49EW1 cyst formation was not explained by the major differences in cyst size, and B1 gene analysis of other organs indicated that low ME49EW1 cyst counts in brain tissue were neither the result of tissue redistribution of cysts nor the use of tachyzoites to infect mice. Secondary ME49EW1 tissue cyst infections also produced very low cyst counts in mouse brain (Fig. 1, second infection). The loss of developmental competency following forced adaptation to HFF cell culture is a common outcome (11). Infection of CBA/j mice with a common laboratory strain (10,000 tachyzoites, i.p.) used to study bradyzoite development *in vitro* (PruQ) (12) also produced far fewer tissue cysts in CBA/j mice at 30 days post-infection (d.p.i.) when compared to native ME49EW parasites (Fig. 1, PruQ-1st inf.). Similar to the ME49EW1 strain, secondary infections with PruQ tissue cysts did not reverse poor cyst production in CBA/j mice (Fig. 1, second infection). These data demonstrate the rapid loss of developmental competency of parasites cultured *in vitro*. How to determine the pathways that are lost and how to study recrudescence need an alternative strategy. Thus, in the absence of a suitable laboratory strain to investigate bradyzoite developmental biology, we optimized the unadapted ME49EW strain to reliably produce 5–10,000 tissue cysts per mouse cortex with yields of 1–2 million purified bradyzoites per mouse [for complete protocols, see methods in reference (10)].

### Mature ME49EW tissue cysts harbor dormant bradyzoites

**Fig 2:**
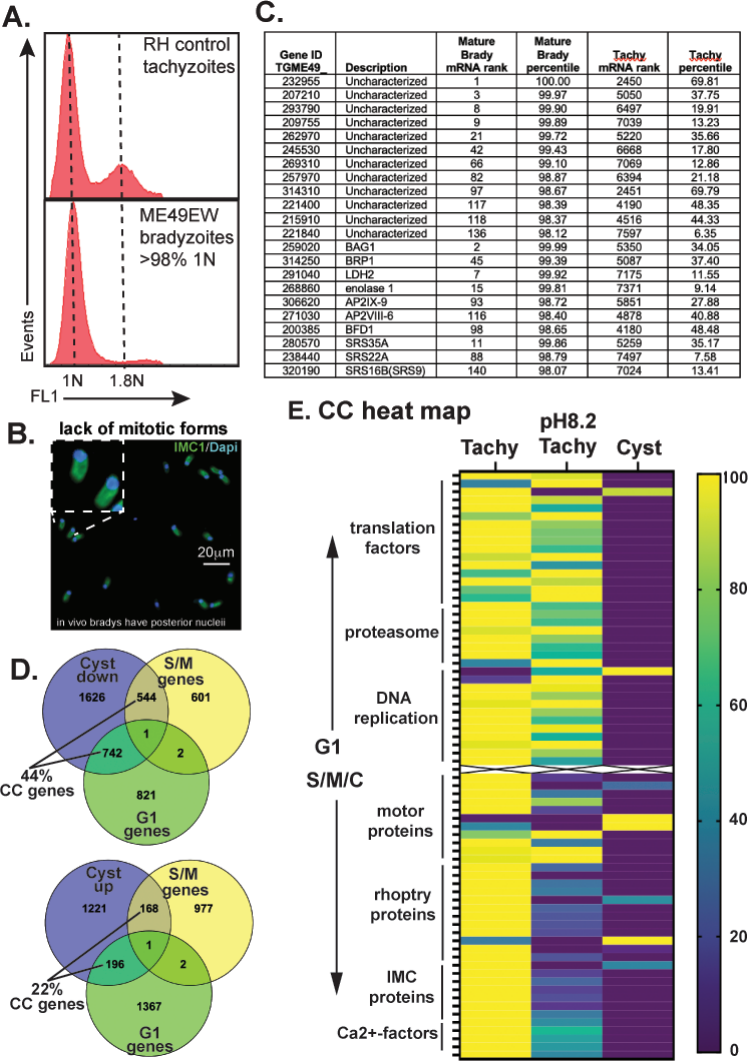
ME49EW bradyzoites in mature tissue cysts are at the dormant growth stage. (A) Genomic DNA analysis of ME49EW excysted bradyzoites (40 d.p.i.) in comparison to Type I RH tachyzoites (representative of two independent repeats). Dashed lines refer to 1N and 1.8N fluorescence peaks in the asynchronous control. DNA fluorescence was measured in FL-1 (x-axis) and 70,000 events (y-axis) were collected for each histogram. (B) Representative images of ME49EW excysted bradyzoites (1,000 × 2 bradyzoites were independently examined) co-stained for IMC1 (budding) and 4′,6-diamidino-2-phenylindole (DAPI) (nuclei). Note the lack of mitotic forms (internal or u-shaped daughters or duplicated nuclei) and the extreme posterior location of the nucleus [see inset image and also reference (14)]. (C) Total RNA-sequencing analysis of ME49EW *in vivo* bradyzoites (40 d.p.i.) vs. *in vitro* ME49 tachyzoites (Database S1). Examples of abundantly expressed transcripts from *in vivo* bradyzoites (upper half of mRNA pool) compared to *in vitro* tachyzoites. Relative mRNA rank and percentile expression are indicated. (D) ME49EW bradyzoite mRNAs twofold higher or lower than ME49 tachyzoite mRNAs were compared to the *Toxoplasma* G1 and S/M reference transcriptomes (16). (E) Heat map comparison of selected G1 and S/M/C cell cycle regulated mRNA expression (16) in ME49 tachyzoites grown in normal or alkaline media vs. *in vivo* ME49EW bradyzoites (see Database S1 for the list of selected cell cycle mRNAs). The mRNA levels of each cell cycle gene were normalized across the three RNA-sequencing data sets.

A recurring question is the degree of dormancy of *in vivo* bradyzoites found within mature tissue cysts. We determined that 98% of ME49EW bradyzoites from the mouse brain (>30 d.p.i.) possessed a 1N genomic content (G1/G0 state) (Fig. 2A), which matches the 1N genome content we determined for Type III VEG strain bradyzoites and sporozoites (13). The genome analysis was independently confirmed by the lack of cell biological evidence of daughter budding or duplicated or u-shaped nuclei in 2,000 bradyzoites examined (2 × 1,000 independently counted). Moreover, all bradyzoites had a posterior nuclear location (Fig. 2B), which was also reported in a recent study of nucleolus factor NF3 (14), that is likely incompatible with active mitosis. Altogether, the nuclear features of mature ME49EW bradyzoites are consistent with the economy of growth dormancy. Total RNA sequencing of bradyzoites from *in vivo* ME49EW tissue cysts was used to establish the bradyzoite transcriptome of a strain that has not been adapted to cell culture and to provide a starting point for analyzing mRNA expression in bradyzoite recrudescence. ME49EW tissue cysts were isolated from CBA/j mice, total RNA was purified, and cRNA libraries were prepared. Illumina sequencing of ME49EW cyst RNA yielded >50 million total reads with 13.8% aligned to the ME49B7 *Toxoplasma* reference genome. We also extracted and sequenced total RNA from *in vitro* ME49 tachyzoites grown in normal (~33 million reads/81% aligned) or alkaline media (~32 million reads/82% aligned) (see Database S1). RNA-sequencing results (FPKM ≥ 1) from each parasite source were ordered from high to low expression (mRNA rank number #1 = highest expressed) and percentile expression, and the relative size of the mRNA molecular pool was determined (Database S1). A total of 6,593 and 6,809 transcripts were identified from *in vivo* bradyzoites and *in vitro* tachyzoites, respectively. Consistent with the lower rates of replication, estimates of mRNA pool size (total FPKM) indicated that the mRNA pool in bradyzoites was ~200% smaller than that of tachyzoites. As found previously for *Toxoplasma* transcriptomes (15), *in vivo* bradyzoites and *in vitro* tachyzoite transcriptomes were lower in complexity with 2–3% of the highest expressed mRNA species (percentile expression >97%) comprising 50% of the total mRNA molecular pool. The mRNA species composition of the upper half of the mRNA pool in bradyzoites (216 mRNAs) and tachyzoites (195 mRNAs) revealed clear biological differences (Fig. 2C; Database S1). Established bradyzoite markers (BAG1, BRP1, LDH2, and ENO1), a number of developmentally regulated SRS-related surface proteins including SRS9, and several ApiAP2 and BFD1 transcription factors were abundantly expressed mRNAs in bradyzoites (Fig. 2C). A total of 26 mRNAs encoding ApiAP2 factors were expressed above the 80th percentile in bradyzoites as compared to only six ApiAp2 factors in tachyzoites (Database S1). Compared to tachyzoites, an increased expression of transcripts encoding uncharacterized (UNC) proteins was a feature of *in vivo* bradyzoites. More than twice as many UNC proteins were included in the top half of the bradyzoite mRNA pool and overall, 33 UNC mRNAs were expressed 50-fold higher in bradyzoites compared to eight UNC mRNAs specific for tachyzoites, highlighting the biology still to be uncovered within the bradyzoites (Fig. 2C and UNC lists; Database S1). Consistent with growth dormancy, twice as many *in vivo* bradyzoite mRNAs (using a ±twofold cutoff) were downregulated (2,916 mRNAs) than upregulated (1,585 mRNAs) (Fig. 2D; Database S1) in comparison to tachyzoites. Nearly half of the downregulated bradyzoite mRNAs (44%) were identified as G1 or S/M cell cycle regulated mRNAs of replicating tachyzoites (16). The RNA-sequencing results for selected cell cycle regulated mRNAs that have peak expression in G1 vs. S and mitotic phases demonstrated the dramatic downregulation of cell cycle mRNAs in ME49EW bradyzoites compared to tachyzoites grown in standard or alkaline-media conditions (Fig. 2E). Tachyzoites stressed by alkaline media expressed higher levels of G1 transcripts, which is the result of the lengthening of the G1 period in these slower growing parasites (13, 17).

### ME49EW bradyzoites recrudesce into fast-growing tachyzoites

To establish a model of ME49EW bradyzoite recrudescence, we investigated *ex vivo* bradyzoite infections of two principal host cells, HFF cells and primary mouse astrocytes. The HFF host cell has been used to study *Toxoplasma* development for over 100 years and has allowed the standardization of many genetic and cell biological techniques. Primary astrocytes, which are relatively easy to culture, are a major host cell that bradyzoites encounter during recrudescence in brain and eye tissues (5, 18, 19). To mimic the invasion-growth-reinvasion process of natural parasite infections and to allow for continuous tracking of the growth rate and developmental changes, astrocyte or HFF cell cultures were infected with *ex vivo* bradyzoites and passed on Day-3, Day-5, and Day-7 (Fig. S1A). A similar strategy was used to successfully characterize *Toxoplasma* sporozoite-initiated development (20).

**Fig 3:**
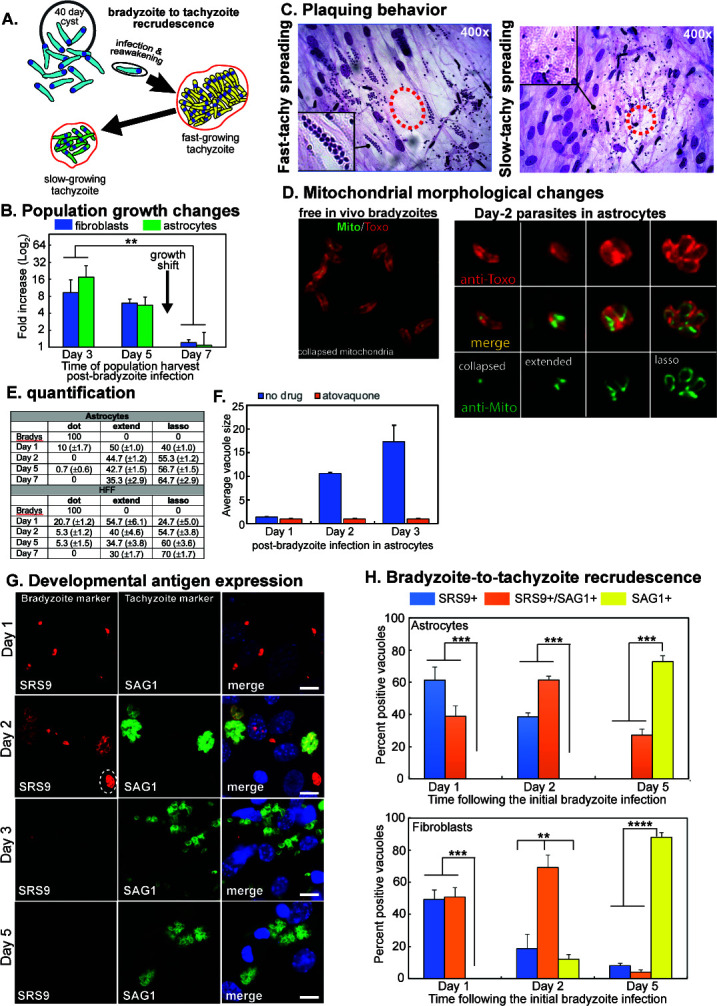
Bradyzoite recrudescence initiates distinct patterns of parasite growth. (A) Diagram of *ex vivo* bradyzoite recrudescence into two sequential tachyzoite stages. (B) Representative population growth (independently measured three times) during three growth periods in HFF (blue bars) or astrocyte (green bars) monolayers (see growth period definitions in Fig. S1A). Note the significant spontaneous growth reduction after Day-5 (**P < 0.01, Day-3 vs. Day-7). (C) Representative lytic patterns of Day-3 and Day-11 populations in HFF cells (first and fourth growth periods). Dashed circles indicate the approximate center of each plaque. Inset images show higher magnification of equal areas of the plaque; note the nuclei size differences and relative MOI differences in each plaque. (D) Representative images of excysted ME49EW bradyzoites and Day-2 parasites co-stained with anti-mitochondria and anti-*Toxoplasma* antibodies. (E) Quantitation of the three major transitional mitochondrial morphologies (collapsed, extended, and lasso) observed during *ex vivo* bradyzoite recrudescence (3 × 50 vacuoles selected at random). (F) The addition of atovaquone (100 nM) 4 h post-bradyzoite infection of astrocytes completely blocked recrudescence and parasite growth (all single-bradyzoite vacuoles) over the 3-day experimental timeframe, while untreated cultures recrudesced and grew normally. Vacuole size was determined by counting 3 × 50 vacuoles selected at random. (G) Representative images of parasite staining patterns on Day-1 and Day-2 (first monolayer) and Day-5 (second monolayer) from the original ME49EW bradyzoite infection of astrocytes (see Fig. S2 for infected HFF images). Infected astrocytes were fixed and co-stained for SAG1 (green) and SRS9 (red) with DAPI staining included as a reference (DNA, blue). Note that the parasites appear rounder in astrocytes than in HFF cells due to the thicker monolayer. The dashed circle in the Day-2 image is a SRS9-Hi/SAG1-low vacuole, while the other three replicating vacuoles in this microscope field are SRS9-low/SAG1-Hi. Day-5 parasites are primarily SAG1+. Scale bars = 10 µm. (H) Quantification of SAG1+ and SRS9+ only expression as well as parasites co-expressing these antigens are shown in panel (G) and Figure S2 (3 x 50 vacuoles selected at random for each time point and host-cell infection). Statistically significant expression of bradyzoite antigen, SRS9+ and SRS9+/SAG1+ vs. tachyzoite antigen, SAG1+, was observed in Day-1 and Day-2 populations, which was reversed in Day-5 populations (**P < 0.01; ***P < 0.001; ****P < 0.0001).

Excysted bradyzoites from *in vivo* ME49EW tissue cysts readily invaded HFF cells and astrocytes and followed a similar course of growth during the first 7-day post-bradyzoite infection (see Fig. 3; Fig. S1 and S2). The majority of bradyzoite vacuoles (Day-0 infection) exhibited delayed replication during the first 24 h in HFF or astrocyte host cells (Fig. S1B and S2). Consistent with the need to reawaken after dormancy, at 24 h following infection, vacuoles containing only single parasites remained the majority in both astrocytes (60% single-parasite vacuoles) and HFF cultures (70% single-parasite vacuoles). This is in contrast to actively growing tachyzoites, where our previous studies established a short window (4 h) for the resumption of cell division following host cell invasion (21). In the next 24 h (Day-1 to Day-2), ME49EW parasite replication quickly accelerated with the majority of vacuoles containing 16 or 32 parasites by the end of Day-2 (Fig. S1B). Qualitatively, parasites growing at these fast rates (~6-h division cycle) possessed a larger nucleus, invaded nearby host cells at high MOI, and the plaques showed a relatively clear lysis zone with less cellular debris (Fig. 3C, Day-3). ME49EW population growth was similar in the first two growth periods (0–3 days and 3–5 days) in both fibroblasts and astrocytes (Fig. 3B). However, 1 week from the initial bradyzoite infection, parasites in either host cell type spontaneously slowed their growth. This was evident by smaller vacuole sizes (Fig. S1B), lower population growth (Fig. 3B), and reduced doubling rates of Day-7 populations (Fig. S1B, Day-7). Slower growth in HFFs and astrocytes became the dominant phenotype of ME49EW populations after Day-7 indicating that the growth shift was host cell independent. Qualitatively, plaque spreading behavior also changed with plaque development similar to many slow-growing laboratory strains, i.e., far fewer multiple invasions of nearby cells, parasites with smaller nuclei (indicative of G1), and cellular debris in a less-defined lysis zone (Fig. 3C, Day-11). Longer cell cycle times in *Toxoplasma* laboratory strains are the result of extended G1 periods (17), which can be estimated from centrosome patterns. ME49EW parasites possessing single (G1 phase) vs. double (S and M/C phases) centrosomes were equal in the faster growing populations (e.g., Day-2, Fig. S1C) and nearly 2:1 in the slower growing populations from Day-7 and Day-9 (Fig. S1C).

The switch from dormant bradyzoite to fast-replicating tachyzoite was associated with dramatic changes in mitochondrial morphology (Fig. 3D and E) (22). The mitochondrial organelle in excysted bradyzoites at Day-0 is a single, collapsed structure (Fig. 3D) but within 48 h this extends and expands to surround the parasite nucleus (lasso form) in Day-2 parasites (Fig. 3E). By Day-5 post-bradyzoite infection, the collapsed form was absent in the infected astrocyte, replaced by a mixture of extended and lasso shapes (Fig. 3E). The timing of the morphological changes indicates that reactivation of the mitochondria is an early event in recrudescence, and in agreement, blocking the function of the mitochondria with the drug atovaquone (23) fully inhibited bradyzoite recrudescence (Fig. 3F). Thus, excysted bradyzoites follow a predetermined pattern of growth involving a reawakening period (24 h) then rapid expansion of parasite numbers, followed by a predictable slow growth period by Day-7 post-excystation. This growth pattern is entirely independent of the host immune response or whether recrudescence is occurring in fibroblasts or astrocytes.

To understand the developmental pathways associated with ME49EW bradyzoite recrudescence, we investigated the expression of two key surface antigens; SRS9 present on bradyzoites and the tachyzoite surface antigen SAG1 (Fig. S1D and E). ME49EW bradyzoites invading host cells were SRS9+/SAG1− in single vacuoles (Fig. S3G). Between Day-1 and Day-2 post-bradyzoite infection, the proportion of parasites expressing SRS9+ began to decline as SAG1 expression increased with a transition observable in some vacuoles that have equal expression of SAG1 and SRS9 (Fig. 3G; Fig. S2). Elevated SAG1 expression progressively became the major phenotype in Day-5 populations as quantified in both host cell types (Fig. 3H).

### Astrocyte host cells support diverse developmental pathways

**Fig 4:**
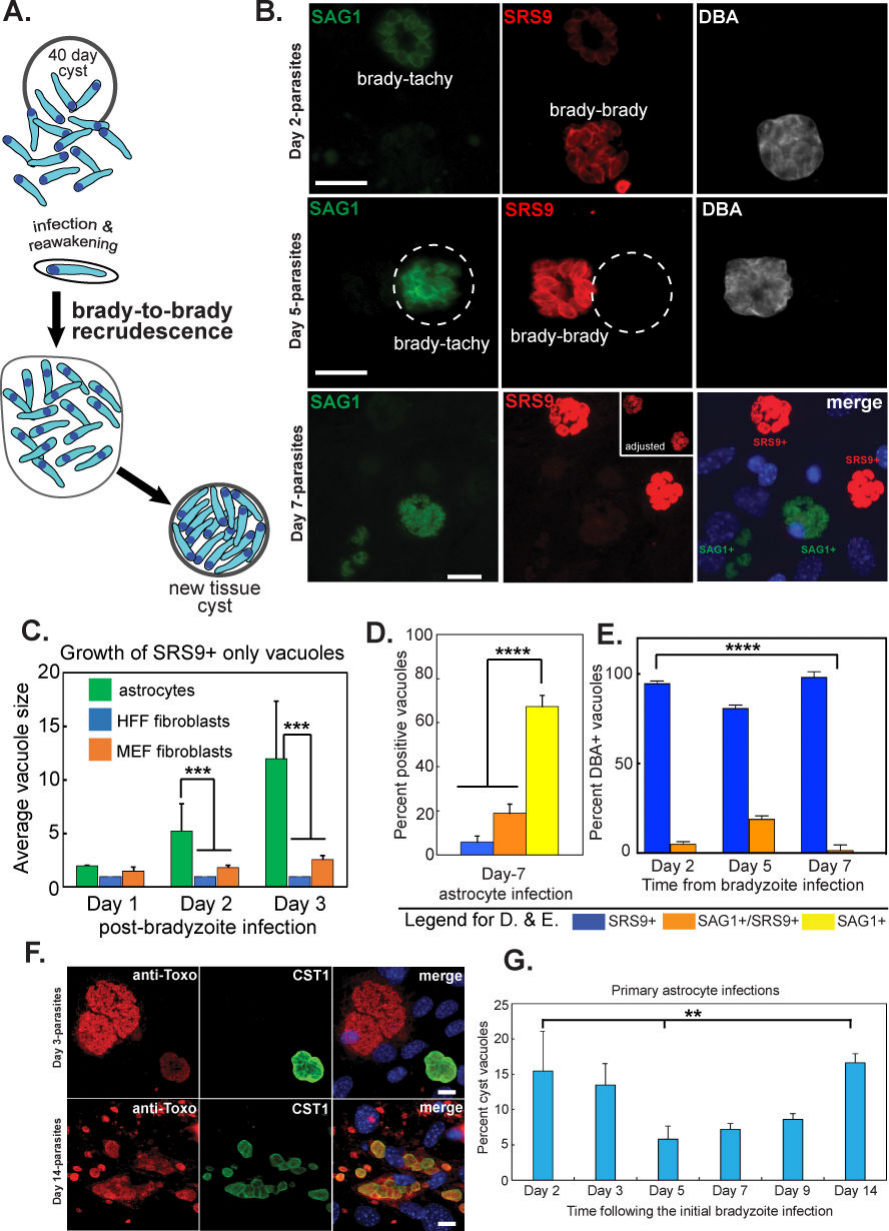
Astrocytes support brady-brady replication. (A) Diagram of brady-brady replication. (B) Top two panels: representative images of parasite vacuoles from Day-2 and Day-5 infected astrocytes. Parasites were co-stained with antibodies against SRS9 (red) and SAG1 (green) and with biotin-labeled DBA to identify vacuoles possessing cyst walls. Bottom panel: Day-7 post-bradyzoite infection, astrocytes were co-stained for SRS9 (red), SAG1 (green), and DAPI (blue, DNA). Host nuclei in the merged image are prominent. Size bars (10 µm) are indicated and the dashed circles indicate the vacuole containing SAG1+ parasites. (C) Growth of SRS9+ only vacuoles over 3 days in HFF and mouse fibroblasts (MEF) vs. primary astrocyte host cells. Vacuoles sizes were determined in 3 × 50 randomly selected vacuoles. Note that in HFF and MEF host cells vacuoles containing SRS9+ parasites showed minimal replication, while SRS9+ parasites were undergoing active replication in astrocytes (Day-2 and Day-3 growth in astrocytes vs. HFF or MEF, ***P < 0.001). (D) Quantification of SRS9+ only, SRS9+ and SAG1+, or SAG1+ only vacuoles in Day-7 populations (3 × 50 vacuoles) from astrocyte cells is shown. SAG1+ only parasites were the dominant population (****P < 0.0001). (E) Quantitation of tissue cyst wall formation (DBA+) as compared to surface antigen expression (SRS9 vs. SAG1) in 3 × 50 vacuoles from randomly selected microscopic fields. Note: No vacuole containing SAG1+ only parasites showed evidence of cyst wall formation and only a small fraction of vacuoles with parasites expressing both SRS9 and SAG1 were found to have cyst walls. Thus, cyst wall formation occurred primarily in vacuoles where parasites only expressed SRS9 at all timepoints examined (****P < 0.0001) (F and G) Representative images of tissue cysts in ME49EW-infected astrocytes at Day-3 (the first growth period) and Day-14 (fourth growth period) from the original ME49EW bradyzoite infection. Anti-CST1 staining (cyst wall, green), anti-*Toxoplasma* (red), and DAPI (DNA, blue). Scale bars = 10 µm. Note The Day-3 image shows a single cyst along side two large vacuoles of rapidly growing tachyzoites. The tight foci containing numerous cysts of different sizes shown in the Day-14 image indicate bradyzoite re-invasion and replication are likely occurring in astrocytes following the growth shift at Day-7. Graph: quantitation of the average percentage of vacuoles (3 × 50 vacuoles randomly selected) containing a tissue cyst wall at various times from the original bradyzoite infection. Growth/monolayer periods: Days 0–3, Days 3–5, Days 5–7, and Days 9–14. Statistically significant cyst wall formation was observed (**P < 0.01) in Day-2 and Day-14 populations vs. Day-5 populations.

The classical and predicted bradyzoite-to-tachyzoite differentiation was not the sole developmental process occurring during ME49EW bradyzoite recrudescence. In addition, we observed SRS9+/SAG1− bradyzoites replicating (brady-brady) in astrocyte cells (Fig. 4A model) simultaneous to tachyzoite replication (SAG1+ only) in neighboring cells (Fig. 4B). The SRS9+-only bradyzoites grew slower (Fig. 4C) progressing from an average vacuole size of 5.22 bradyzoites (SAG1+ tachyzoite vacuole = 10) at Day-2 to an average vacuole size of 12 bradyzoites by Day-3 (SAG1+ tachyzoite vacuole = 21). Neither HFF nor murine embryonic fibroblast cells supported brady-brady replication (Fig. 4C). There were SRS9+/SAG1− vacuoles present beyond Day-1 in HFF cells, which contained single parasites indicating they were non-replicating bradyzoites most likely carried over during passage or growth arrested tachyzoites that re-expressed SRS9, while turning off SAG1. SAG1+-only parasites continued to dominate populations in HFF cells and astrocytes even after the growth of parasites spontaneously slowed (Fig. 4D, Day-7 astrocytes). However, bradyzoite replication continued to persist (7% of Day-7 vacuoles were SRS9+) along with an increase in double-positive vacuoles (SRS9+/SAG1+, 34%) indicating some transitionary parasites (Fig. 4D). Co-staining parasites in astrocytes with biotin-labeled *Dolichos biflorus* agglutinin (DBA) established spontaneous cyst wall assembly that primarily occurred in vacuoles undergoing brady-brady replication (SRS9+ only, Fig. 4E). ME49EW brady-brady replicating vacuoles with cyst walls in astrocytes were 15% of Day-2 vacuoles. As exponential growth of SAG1+ tachyzoites dominates, the relative proportion of cyst vacuoles decreases, followed by a progressive increase after the Day-7 growth shift (see Fig. 4F and G). By Day-14 post-bradyzoite infection, localized clusters of variable-sized tissue cysts were numerous (see examples, Fig. 3F). In contrast to astrocyte infections, we did not detect spontaneous tissue cyst formation in infections of HFF cells.

### Transcriptomics of bradyzoite recrudescence reveals multifunctional development potential

**Fig 5:**
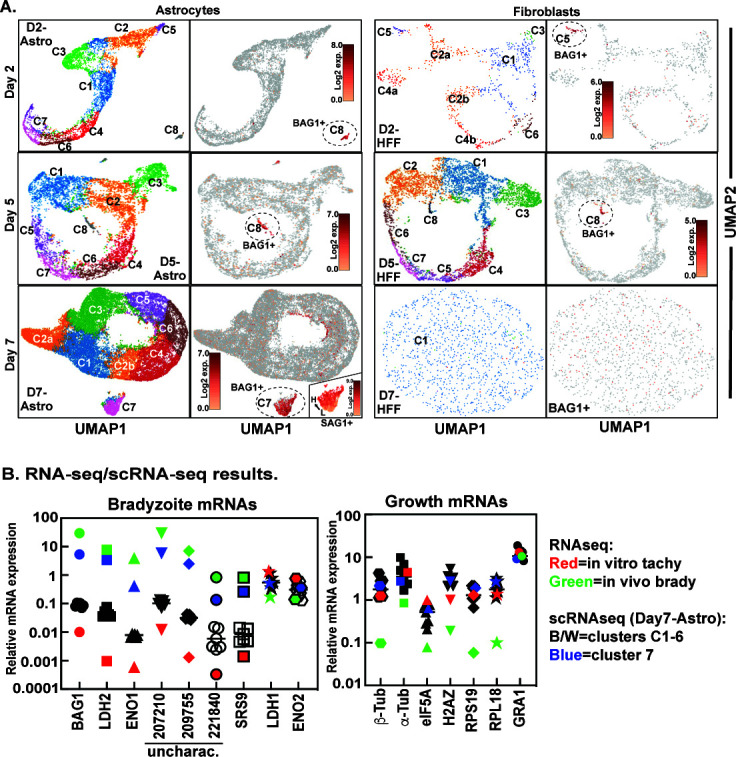
Confirming the complexity of bradyzoite-initiated development by single-cell transcriptomics. (A) ME49EW recrudescing parasites from Day-2, Day-5, and Day-7 post-bradyzoite infections in astrocytes or HFF cells were subjected to single-cell RNA sequencing (scRNA-sequencing, Database S2) and the transcriptome results were visualized by UMAP. Expression shading of the mRNA encoding the bradyzoite-specific heat shock protein, BAG1, onto the UMAP projections (immediately right of UMAP images) distinguishes tachyzoite (BAG1−) from bradyzoite (BAG1+) stages. Distinct bradyzoite clusters are indicated by dashed circles in the UMAP images with the relative levels of BAG1 mRNA expression indicated by a scale bar in each image. (B) Statistically validated mRNA levels from RNA-sequencing and scRNA-seq data were further normalized by the expression of the internal constitutive marker, GAPDH1, permitting the direct comparison of selected bradyzoite specific and growth genes across data sets. Levels of normalized mRNA expression differed by up to seven orders of magnitude, and thus, were plotted on a Log10 scale. Left graph: relative mRNA expression of the canonical bradyzoite markers, lactate dehydrogenase (LDH2) and enolase 1 (ENO1), and other selected bradyzoite genes (UNCs and SRS9) as well as tachyzoite-specific LDH1 and ENO2 mRNAs in scRNA-sequencing clusters (C1–7) from the Day-7 astrocyte sample compared to *in vivo* bradyzoite and *in vitro* tachyzoite populations (total RNA-sequencing data). Note that bradyzoite-specific gene expression levels move in a developmentally coordinated fashion within a scRNA-sequencing cluster or RNA-sequencing sample. C7 parasites from Day-7 infected astrocytes (blue colored marker) had elevated bradyzoite mRNA expression similar to *in vivo* bradyzoite populations (green colored marker), while the expression of these mRNAs from C1–6 tachyzoites (black and white markers) was more similar to *in vitro* tachyzoite populations (red colored marker). Right graph: Expression of selected growth genes in comparison to the constitutively expressed GRA1 gene. Note that with respect to growth gene expression, C7 parasites (BAG1+) were more similar to other tachyzoite stage parasites than to *in vivo* bradyzoites. See Databases S1 and S2 for complete gene lists.

Immunofluorescence analysis demonstrated that *ex vivo* bradyzoite recrudescence in astrocytes involves at least two replicating parasite populations. To further investigate the molecular basis of bradyzoite recrudescence, single parasites from Day-2 (post-brady reawakening), Day-5 (tachy dominant), and Day-7 (brady re-emergent) astrocyte vs. HFF infected cells were captured using the 10× Chromium Single Cell Gel Bead kit and 3′-RNA-sequencing libraries were sequenced. A total of 57,746 cells passed quality controls (Fig. S3A and B) yielding ~6 billion reads with an average of 78% of reads mapping to the *Toxoplasma* genome. The scRNA-sequencing data were embedded using Uniform Manifold Approximation and Projection (UMAP), and parasite transcriptome intersection was determined using graph-based K-means (*k* = 8) clustering (Fig. 5A). To distinguish tachyzoites from bradyzoites, UMAP images were shaded with the levels of BAG1 mRNA expression (TgME49_259020), a small heat shock protein specifically expressed in bradyzoites. Single-cell RNA sequencing of parasites from astrocytes (Day-2, Day-5, and Day-7) confirmed that bradyzoite recrudescence produced a large number of replicating tachyzoites (BAG1− parasites) and tachyzoite replication was also prevalent in the Day-5 sample from HFF cells. These asynchronously growing tachyzoite populations assembled into cyclical patterns (Fig. 5A) that were similar to the progressive cell cycle transcriptome of synchronized tachyzoites (16). Clusters of tachyzoites expressing the highest levels of G1 transcripts were positioned opposite to the tachyzoite clusters, where S/M/C phase transcripts were maximally expressed in each circular UMAP pattern (see Fig. S3C for cell cycle heat maps).

In contrast to the parasites grown in HFFs, BAG1+ parasites in astrocytes remained viable and supported bradyzoite development (Fig. 4C). Single-cell RNA sequencing of Day-7 parasites from astrocytes identified a large group of BAG1+-positive bradyzoites in transcriptional cluster 7 (1,098 cells), which were distinct from the coordinated cell-cycle transcriptomes of BAG1− tachyzoite clusters C1–6 (Fig. 5A, D7-Astro). Cluster 7 accounted for the majority of parasites expressing elevated BAG1 mRNA in this sample (Fig. 5A, D7-Astro BAG1 shading), and furthermore, these BAG1+ parasites expressed increased levels of other canonical bradyzoite genes along with bradyzoite-specific UNC mRNAs and mRNAs encoding brady surface antigens such as SRS9+ (Fig. 5B, blue markers). Cluster 7 parasites expressed variable levels of SAG1 mRNA, ranging from near zero- to sixfold higher mRNA levels (Fig. 5A, D7-Astro-inset SAG1 shading), which was consistent with a mixture of replicating bradyzoites and tachyzoites transitioning to bradyzoites following the growth shift in Day-7 astrocytes. The expression of bradyzoite-specific mRNAs in cluster 7 parasites was similar to *in vivo* bradyzoites from tissue cysts (Fig. 5B, blue and green markers); however, the scRNA-sequencing data from cluster 7 bradyzoites were not consistent with the growth dormancy of *in vivo* bradyzoites (Fig. 2E). Cluster 7 parasites had a G1 enhanced mRNA profile similar to alkaline-stressed parasites, which was consistent with parasites growing at a slower rate (compare Fig. 2E to Fig. S3C). The genes encoding alpha and beta-tubulin, translation initiation factor 5A, and ribosomal proteins L18 and S19 are sentinel growth markers that show reduced mRNA expression in mature cyst bradyzoites (Fig. 5B, green markers). Genetic deletion studies in yeast demonstrated unique cell cycle roles for ribosomal proteins L18 (S/M phase) and S19 (G1 phase) (24) that may have been preserved in *Toxoplasma*. Importantly, mRNAs encoding these sentinel growth markers were similar in all Day-7 parasites from astrocytes including the bradyzoites in cluster 7, and were different from *in vivo* dormant bradyzoites (Fig. 5B). Smaller numbers of bradyzoites in Day-2 and Day-5 samples from astrocytes followed the bradyzoite-specific and G1-enhanced transcription pattern of Day-7 bradyzoites (Fig. S3C and D). There were qualitative differences between astrocyte and HFF parasite samples with fewer parasites from HFF cells passing QA controls (Fig. S3B). Nonetheless, it was possible to establish the cell cycle profile of tachyzoites from Day-5 HFFs (Fig. S3C) and small distinct bradyzoite clusters were detected in Day-2 and Day-5 HFF samples, although not in the Day-7 sample (Fig. 5A). Altogether, the transcriptional features of parasites from astrocytes validate the unique relationship of brady-brady replication occurring alongside brady-tachy recrudescence within the astrocyte host cell.

### Bradyzoites and recrudescing populations have distinct capacities to form tissue cysts in mice

**Fig 6:**
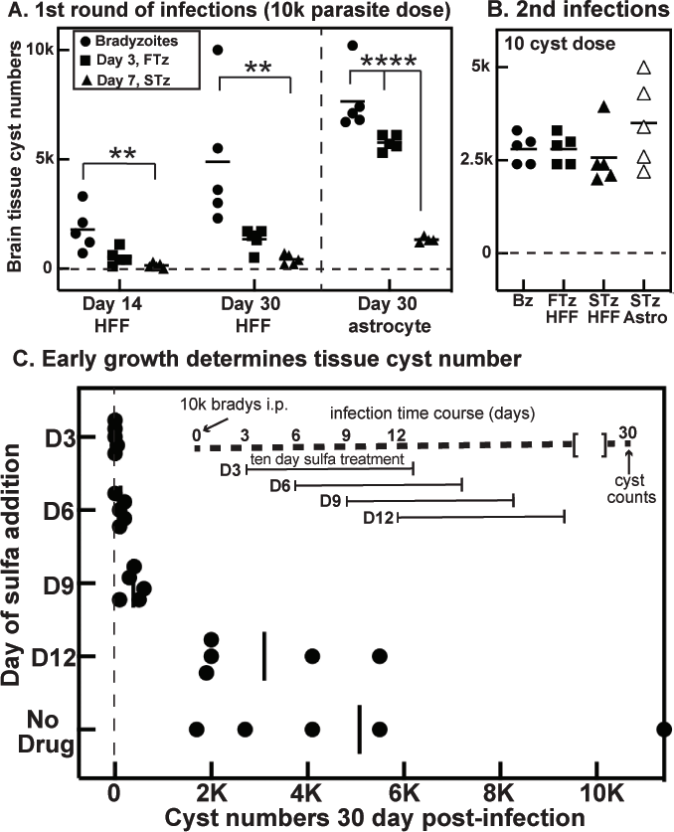
Bradyzoite recrudescent stages have distinct functions in mice. *In vivo* ME49EW bradyzoites were obtained from tissue cysts purified from mouse brain tissue (closed circles). Bradyzoite infection of HFF or astrocyte cells was used to obtain Day-3 parasites (fast-growth tachyzoites, FTz, closed squares). Passage of Day-5 parasites into HFF or astrocytes provided Day-7 tachyzoites (slow-growth tachyzoites, STz, closed triangles). (A) Groups of five CBA/j mice (45 total) were used to evaluate tissue cyst numbers in brain tissue at 14 or 30 d.p.i. Infections: 10^4^ bradyzoites/mouse inoculated i.p., with cyst counts at 14 and 30 d.p.i. (two independent cyst preparations); 10^4^ Day-3 FTz-parasites inoculated i.p., with counts at 14 (only HFF-derived parasites) and 30 d.p.i. (HFF- or astrocyte-derived); 10^4^ Day-7 STz-parasites inoculated i.p., with counts at 14 (only HFF-derived) and 30 d.p.i. (HFF- or astrocyte-derived). Statistical significant differences in cyst counts are indicated; **P < 0.01 and ****P < 0.0001. (B) Tissue cysts from Day-30 d.p.i., mice infected in (A) with bradyzoites, FTz-parasites, or STz-parasites (HFF- or astrocyte-derived is indicated) were used to infect new groups of CBA/j mice (five mice/group, 25 total); 10 cysts inoculated i.p., with tissue cyst counts at 30 d.p.i. (C) Five groups of five CBA/j mice (25 total) were infected with 10^4^ purified *in vivo* ME49EW bradyzoites. The times of sulfamerazine (sulfa) addition and duration of treatment are indicated. Tissue cyst counts were determined at 30 d.p.i. Note: Cyst counts when sulfa was added to drinking water at 3 d.p.i. were >400-fold lower than no drug control vs. 13-fold lower when the drug was added at 9 d.p.i. Statistically significant differences in cyst counts in comparison to no drug control were detected for Day-3, Day-6, and Day-9 drug treatment additions (*P* < 0.001).

**Fig 7:**
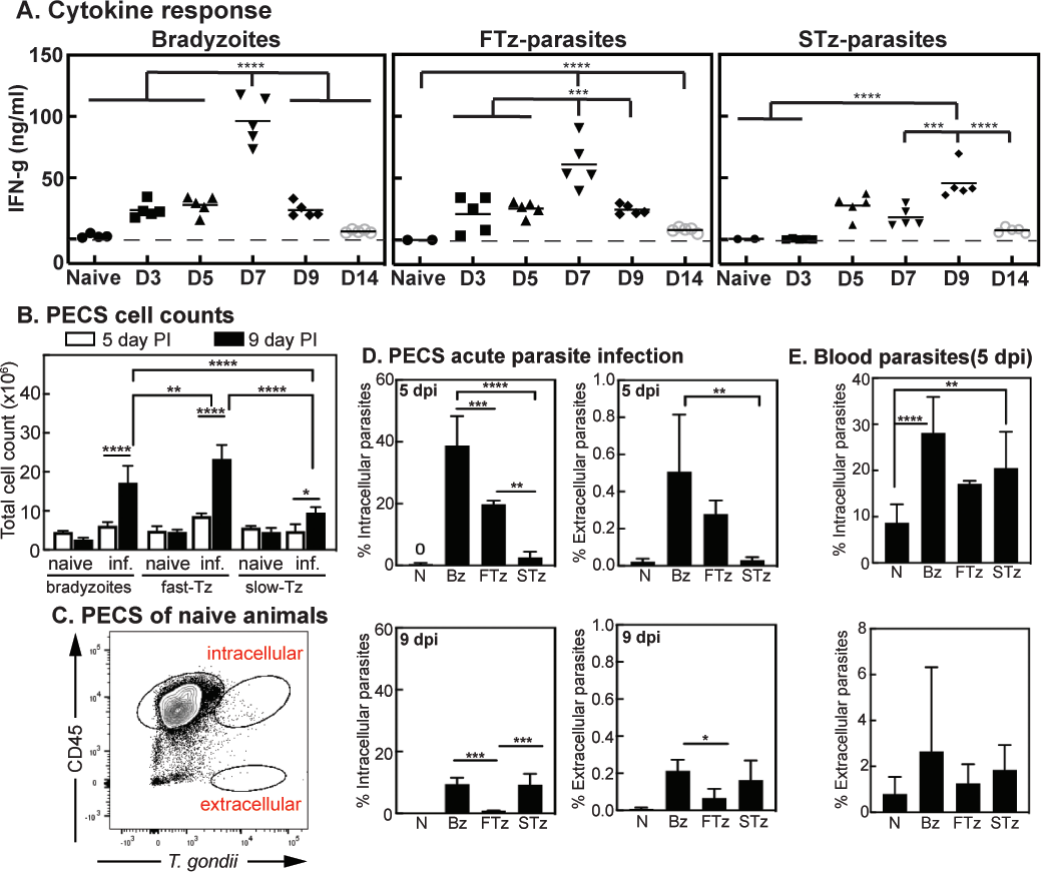
Analysis of ME49EW acute phase infections. (A) Serum was collected from infected animals from Fig. 6 at 3, 5, 7, 9, and 14 d.p.i. with native bradyzoites, FTz-parasites (Day-3 HFF), or STz-parasites (Day-7 HFF), and systemic levels of IFNγ were quantified. Statistical significances (*P < 0.05, **P < 0.01, ***P < 0.001, and ****P < 0.0001) are indicated. Note also that peak IFNγ levels in the bradyzoite infection are significantly higher (*P* < 0.02) than in the STz-parasite infection. (B) Cell counts from PECS of mice infected with ME49EW bradyzoites, FTz-parasites, or STz-parasites. Note the higher cell recruitment to FTz-parasite infections. (C) Flow cytometry layout of naive PECS sample to show gating strategy. (D) Analysis of ME49EW acute infection results demonstrates differences in infection and dissemination capability of bradyzoites, FTz-parasites, and STz-parasites. Quantification of PECS flow results. Significance was determined via one-way ANOVA with multiple comparisons (*P*-value < 0.05). Note the greater decline of intracellular parasites from FTz-parasite infections suggesting increased dissemination. (E) Flow cytometry quantification results of blood taken at 5 d.p.i. from mice infected with parasites from (C) above.

To determine the function and establish the importance of the programmed growth stages following brady-tachy recrudescence, we tested the tissue cyst-forming capability of the ME49EW Day-3, fast-growing (FTz) and Day-7, slow-growing (STz) parasites in comparison to excysted bradyzoites from *in vivo* tissue cysts. CBA/j mice were infected with 10,000 parasites i.p. and tissue cysts were quantified at 14 and/or 30 d.p.i. (Fig. 6A). Excysted ME49EW bradyzoite infections (Fig. 6A, closed circles) produced the highest number of brain cysts indicating intact cysts are not required to achieve robust cyst numbers in the murine brain. Day-3 FTz-parasites (closed squares) also produced reasonable cyst numbers, although the cyst count from HFF-derived parasites was not as high as the paired bradyzoite, FTz-, and STz-populations derived from astrocytes. This difference could be experimental or biological, and will require further investigation to resolve. Regardless of the host cell used, infections with Day-7 STz-parasites resulted in significantly lower cyst numbers in CBA/j brain tissue. Unlike cell culture-adapted parasite strains (Fig. 1), the reduction in cyst formation of ME49EW STz-parasites was not permanent as infections with 10 cysts derived from the brains of the first round of mouse infections gave equivalent cyst numbers irrespective of the parasite’s source of the original infection (Fig. 6B). Relative parasite burden in the brain of these mice correlated well with the cyst count data (Fig. S4A and B), while other tissues had detectable B1 gene levels that were a fraction of the levels in the brain indicating that changes in tissue distribution could not explain the cyst count differences. Serum cytokine responses are a helpful readout of systemic infection and are related to parasite burden; we, therefore, collected blood at various times post-infection and quantified IFNγ levels (Fig. 7A). Peak IFNγ concentrations seen at 7 d.p.i. with bradyzoites and FTz-parasites follow normal kinetics (25), while IFNγ in STz-parasites’ infections confirms successful infection and parasite replication but the reduction in IFNγ concentrations and delay in kinetics were consistent with the significantly lower parasite burden in mice infected with these parasites (Fig. 7A).

To further understand how bradyzoite-initiated recrudescence contributes to tissue cyst formation, we infected CBA/j mice with ME49EW excysted bradyzoites from mice and then blocked parasite growth by adding sulfamerazine to drinking water at various times post-infection (Fig. 6C, diagram). Groups of five mice were infected with 10,000 bradyzoites and subjected to sulfamerazine treatment for a period of 10 days starting 3 days post-infection and continuing until day 12 post-infection. At 30 d.p.i., the brain cyst numbers were determined (Fig. 6C). The addition of sulfamerazine to drinking water at 3 d.p.i. effectively reduced tissue cyst numbers by >400-fold over no drug controls, while addition at 9 d.p.i. reduced average cyst numbers by 13-fold. Treatment started at 12 d.p.i. had no significant effect on average cyst numbers in 30-day infected mice. These results were consistent with the finding that bradyzoites and FTz-populations that formed early in bradyzoite recrudescence have the greatest capacity to produce brain tissue cysts in CBA/j mice (Fig. 6A). Importantly, these results also demonstrate that the complete series of steps in this developmental pathway, which cannot be duplicated by HFF-adapted strains, is likely critical to achieving high cyst numbers in mouse brain tissue.

To determine if the reduced number of cysts in the brain is a result of poor infection and dissemination early during infection, we compared infection parameters at two different acute time points (Day-5 and Day-9; Fig. 7). To quantify infected cells, peritoneal exudate cell suspension (PECS) was analyzed by cell counts (Fig. 7B) and flow cytometry (Fig. 7C and D; Fig. S4C). Using this method, it is possible to identify infected cells and extracellular “free” parasites (26). Results revealed a distinct population of intracellular parasites (CD45+Toxo+) as well as a small but defined population of extracellular parasites (CD45−Toxo+) present in the PECS at both 5- and 9-day post-bradyzoite infection (Fig. 7D; Fig. S4C). The intracellular parasite population decreased step-wise in both FTz- and STz-infections at 5 d.p.i. (Fig. 7D). At 9 d.p.i., FTz-parasites showed significantly less intracellular parasites compared to bradyzoite and STz-parasite infections, which were comparable (Fig. 7D). B1 gene analysis of PECS confirmed significantly lower parasite burden in STz-infection compared to both bradyzoite and FTz-infections at 5 d.p.i., suggesting a defect in the infection with STz-parasites (Fig. S4D). Additionally, parasite burden in the PECS significantly decreased in bradyzoite and FTz-infections between 5 and 9 d.p.i. suggesting possible dissemination and/or immune clearance (Fig. S4D). To test the presence of infection in circulation, flow cytometry was also performed on blood (Fig. 7E). Extracellular parasites, previously detectable in the blood (26), could not be detected above background levels with any infection, which is likely due to lower infection dose. However, both bradyzoite and STz-infections at 5 d.p.i. exhibited significantly elevated percentages of circulating infected cells compared to background. FTz-infection showed no significant increase in intracellular parasites in the blood at 5 d.p.i. indicating possible early dissemination to other tissues such as lung (Fig. 7E). By 9 d.p.i., no intracellular parasites were found above background levels in the blood in any infection suggesting successful dissemination to other tissues (Fig. 7E). Indeed, B1 gene analysis of brain and lung tissues suggests that FTz-parasites have disseminated to peripheral organs within 5 days (e.g., lung, Fig. S4D). Bradyzoite and FTz-infection demonstrated comparable B1 gene signals in brain tissue, and both were significantly elevated compared to STz-infection at 9 d.p.i. In lung tissue, bradyzoite infection showed the highest B1 gene signals with FTz- and STz-infections leading to significantly lower parasite burden. Collectively, these results indicate that bradyzoite and FTz-parasites are capable of successful infection and dissemination during acute infection, and both are able to make it to the brain by 9 d.p.i. despite an enhanced immune response in the PECS. These results also suggest unsuccessful infection and dissemination capabilities of STz-parasites during acute infection, which may explain lower cyst burdens later on during infection (see Fig. 6A).

## DISCUSSION

The molecular basis for why some *Toxoplasma* strains produce thousands of tissue cysts in mouse brain while other strains produce a couple of dozen is not known, but it has been a bottle neck in advancing our understanding of tissue cyst biology. HFF-adapted PruQ parasites slow growth in pH 8.2 media and robustly assemble cyst walls in 48–72 h cell cultures. However, where it really counts in *Toxoplasma* infections is that the PruQ strain differs from the ME49EW strain by two orders of magnitude in the ability to produce tissue cysts in mice (Fig. 1). The paradox of PruQ strain robustly producing cyst walls when stressed by alkaline media in cell culture, while at the same time poorly forming tissue cysts in mice has been reported by a number of laboratories [recently cataloged in reference (27)] and is the consequence of repeated passage in HFF cells. Comparing HFF-based models of bradyzoite development to the spontaneous development of ME49EW cysts in astrocytes raises further concerns. In response to alkaline stress, HFF-adapted parasites remain quasi-tachyzoite as their dominant SAG1+ phenotype reveals, and they display extreme vacuole asynchrony with or without the assembly of cyst walls (28). Here, we show that spontaneous ME49EW bradyzoite development in astrocytes is synchronous within the vacuole, where cyst walls primarily form when SRS9 is uniformly induced and SAG1 is coordinately downregulated. These results highlight the limitation of inferring bradyzoite functionality only from cyst-wall formation, which was first recognized nearly 30 years ago (29) and even more recently (30).

Native bradyzoites are the versatile, multifunctional stage with pluripotent capacity to differentiate into the three asexual replicating stages of the *Toxoplasma* life cycle; brady-to-brady, brady-to-tachy, and brady-to-merozoites. They initiate the definitive life cycle by converting to merozoites in feline epithelial cells or recrudescing into tachyzoites in a variety of host cells leading to reactivation of acute toxoplasmosis in immune-compromised hosts. Studies spanning 30 years support the concept that the cell cycle end point of tachyzoite-to-bradyzoite development is dormancy (13, 31, 32), which is substantiated by our study. ME49EW bradyzoites excysted from mature *in vivo* tissue cysts (>30 d.p.i.) are deeply growth arrested; they have primarily 1N genomic contents (98%), lack detectable mitotic and budding forms, and are slow to resume replication after invasion. Here, we use a ME49 strain that has been passaged continuously *in vivo* for over 20 years between susceptible and resistant strains of mice (10). This protocol is important in generating reliable virulence and cyst number and deviations may result in selecting the parasites incapable of generating the fully dormant bradyzoite potentially generating parasites with increased proliferative capacity within the cyst (32). Comparisons of these strains may reveal further biology in the fully mature bradyzoite.

### Pre-programmed parasite development

Once awakened by cyst disruption and host cell invasion, the development initiated by ME49EW bradyzoites follows a similar pathway that we previously described for sporozoite infections (15, 20). ME49EW bradyzoites sequentially develop into two types of replicating parasites; a fast-growing parasite that switches in days to a slow-growing parasite. ME49EW bradyzoites occupy single parasite SRS9+ SAG1− vacuoles for ~24 h and it takes ~5 days to fully transition to majority SAG1+ tachyzoites. The switch from non-growing bradyzoite to fast-growing tachyzoite required reactivation of the mitochondria, which is a bradyzoite vs. tachyzoite difference that others have noted (33), and fast growth was transient suggesting there may be a finite resource limiting this developmental transition. The amylopectin content of *in vivo* bradyzoites that tachyzoites lack is a prime candidate to investigate as has been suggested in reference (34). The rapid differentiation of ME49EW bradyzoites into a fast-growing tachyzoite tracked with the same timeframe in both fibroblasts and astrocytes as did the shift to slower growth at 6–7 days indicating these growth changes are host cell independent and pre-programmed. The fast-growing ME49EW tachyzoites that can only be obtained from *ex vivo* ME49EW bradyzoite infections *in vitro* are an important new resource for producing transgenic, *in vivo* tissue cysts (10).

### Host cell-dependent parasite development

The new *ex vivo* model of bradyzoite recrudescence confirmed that an alternative brady-brady replication pathway exists in the intermediate life cycle that had been inferred by other research (35, 36). Bradyzoites that invaded primary astrocytes exhibit two forms of replication simultaneously that follow the 24-h reawakening period: brady-tachy conversion and replication (~85% vacuoles) and brady-brady replication (~15% vacuoles). Our results revealed that astrocytes are permissive for brady-brady replication, while fibroblasts are resistant as evaluated in two mammalian sources of this cell type. The scRNA-sequencing of recrudescing parasites in astrocytes confirmed the persistence of bradyzoites through the 7-day period we sampled the recrudescent populations. Collectively, the cell biology and scRNA-sequencing data indicate that this group of parasites is composed of replicating bradyzoites as well as spontaneous conversion of tachyzoites back to the bradyzoite stage after the growth shift at Day-7. Preliminary studies show that replicating bradyzoites (SRS9+/SAG1−) can be purified and grown within tissue cysts for long periods in astrocytes (Ulu and Wilson, personal communication) indicating bradyzoite growth alone can enlarge tissue cysts.

For close to 100 years, studies of the *Toxoplasma* tachyzoite have been conducted using *in vitro* cultured fibroblasts; indeed, the ease of this culture system facilitated the study of *Toxoplasma* tachyzoites as a model for apicomplexan biology. In contrast, our new *ex vivo* model of bradyzoite recrudescence captures two distinct pathways, one host cell dependent and the other independent. Host cell dependency rather than immune-stress induction has been the key feature of *Toxoplasma* development since the discovery of spontaneous cyst development occurring in neurons and myocytes (37). Yet, the heterogeneity achieved in astrocyte cultures of tachyzoite and bradyzoite replication is new and suggests a major portion of the life cycle that has been overlooked. *Toxoplasma* encounters many cell types during its lifecycle, some of which induce cyst production and some that facilitate dissemination. Recrudescence most commonly occurs at three different sites: (i) in the gut following ingestion of tissue cysts, where parasites encounter gut epithelial cells followed by innate immune cells, (ii) in peripheral organs such as the muscle and liver, and (iii) in the brain/central nervous system (CNS) containing neurons, astrocytes, microglia, and, during infection, peripheral immune cells. Determining what characteristics of the astrocyte support brady-brady replication and whether such characteristics exist in other cell types the parasite encounters will have important implications for managing the amplification of bradyzoites and the development of cysts.

### Immune-independent heterogeneity

**Fig 8:**
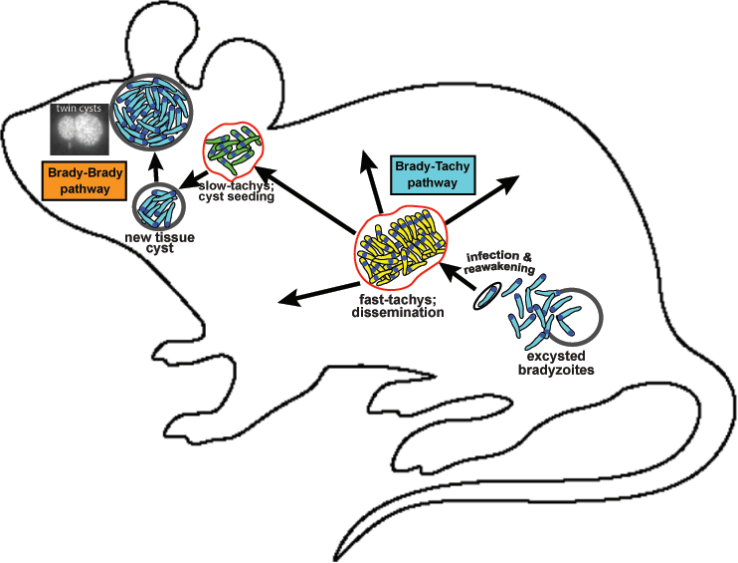
A model of developmental pathways initiated by the bradyzoite stage in the intermediate host. Pre-programmed pathways controlling bradyzoite recrudescence into tachyzoites are independent of host cell type, starting with a reawakening phase (first day), progressing to a fast-replicating phase (2–6 days), and finally, coordinate slowing of population growth (>day 6). Bradyzoite replication is host cell restrictive and likely responsible for cyst expansion and long-term cyst stability in brain tissue.

The study presented here documents bradyzoite replication in astrocytes in the absence of cytokines, particularly IFNγ, or other stress-induction mechanisms. The cytokine IFNγ is critical for protection against toxoplasmic encephalitis (TE) in part by limiting parasite replication in astrocytes (38, 39); indeed, in the absence of IFNγ signaling, cyst formation is observed in astrocytes (19, 39). Our *ex vivo* model by no means solves the question of what prompts a cyst to reactivate; however, in the context of TE (absence of IFNγ) and during initial infection, the developmental pathway uncovered by this *ex vivo* model suggests that the parasite is pre-programmed to undertake two important functions: (i) amplify parasite burden to maximize dissemination and (ii) seed tissues with cysts that will continue transmission (see the model in Fig. 8). These functions occur non-sequentially driven by immune responses but are genetically pre-programmed hence cyst formation even in the absence of IFNγ. Recrudescence is, therefore, a point in the lifecycle in which *Toxoplasma* has the potential to go from a homogeneous to a heterogeneous population composed of both replicating bradyzoites and tachyzoites in the same environment. We propose that this heterogeneity is functional. Brady-brady replication allows for the growth and maintenance of the cyst population either in neurons, resulting in larger cysts and “twin cysts,” or via astrocytes as an energy-rich vessel, facilitating optimal bradyzoite replication and local cyst dissemination (Fig. 8).

### The importance of the full life cycle

Although significant progress has been made using genetically modified lab strains to understand transcriptional regulation, cyst location, number, and kinetics *in vivo*, in the absence of the complete recrudescence pathway (quickly lost *in vitro*), these studies are likely testing the initial seeding of cysts in the brain. Our results show that the complete bradyzoite-to-tachyzoite recrudescence pathway that unfolds over 1 week following purified bradyzoite infections is required for robust expansion, dissemination, and establishing tissue cysts in brain tissue. This window of opportunity is consistent with a recent study showing maximum tissue cyst formation in pig muscle is reached 7 days after oral infection with tissue cysts from mice (40). Infections with the three growth forms of the excysted ME49EW bradyzoite paint a picture of recrudescence following tissue cyst infection. Efficient bradyzoite infection of cells is followed by a rapidly dividing phase after 24 h leading to almost 40% of cells infected by day 5 and a measurable number of extracellular parasites disseminating in the blood and migrating to tissues, with the lung showing early signs of infection. For the next 4 days, fast-growing tachyzoites are seeding tissue, especially the brain at which point they will encounter neurons encouraging cyst formation. By this time, high systemic IFNγ activates host cells to limit parasite replication and clear this lytic form of infection. The pre-programmed switch to a slower-growing tachyzoite stage by days 7–9 enhances both the immune clearance of the acute infection and promotes conversion to the bradyzoite stage. This timing is supported by our data showing that drug treatment after day 9 is ineffective in limiting cyst burden confirming that the slowing of parasite growth effectively ends dissemination. If the complete pathway is not intact and the fast-growing tachyzoite phase is skipped, as is the case when we infect with slow-growing tachyzoites, then cysts in the brain are >50-fold lower than infection with purified bradyzoites. The viability of these cysts is intact demonstrating that the parasites are cyst-competent. Instead, it is the slow growth and poor dissemination of this slow-growing stage of the recrudescent pathway supported by fewer infected cells and a delay and reduction in the magnitude of the host response that likely account for low cyst numbers. We postulate that many of the lab-adapted strains that remain cyst-competent are stuck in this slow-growth phase of replication. In contrast, purified bradyzoites that remain single undivided parasites for 24 h result in a greater cyst burden in the brain over the rapidly dividing tachyzoites. Of note, the bradyzoite infections exceed the fast-growth tachyzoite in almost every parameter except the number of cells recruited to the site of infection. This could suggest a “stealth-like” component of the excysted bradyzoite stage, which would be missed in the rapidly dividing and lytic (immune-amplifying) fast-growth tachyzoite infections or an enhanced capacity to manipulate host cell migration thereby increasing dissemination as has been documented for tachyzoites (41).

In summary, the dormant bradyzoite in ME49EW tissue cysts possesses remarkable developmental flexibility and the ability to tailor the parasite-encoded developmental program to the type of host cell infected (definitive or intermediate host), which is fully captured by the application of cell biology and scRNA-sequencing in a new *ex vivo* astrocyte recrudescence model. In the right host cell, ME49EW parasites can choose to replicate as bradyzoites or fully convert to the tachyzoite stage in order to disseminate. Native bradyzoites, like the sporozoite stage, develop into the tachyzoite stage using a two-step pathway that permits rapid biomass expansion but is also self-limiting. These versatile tachyzoites also have the ability to re-develop into the dormant bradyzoite at any time within a host cell environment that is permissive for tissue cyst development. Bradyzoites likely reinvade neighboring host cells and continue to proliferate, as previously reported for free bradyzoites (36). Adapting native strains to HFF cell culture disrupts these pathways and may cause transcriptional confusion as recent single parasite RNA-sequencing studies have suggested (42).

## MATERIALS AND METHODS

### Parasite strains and host cells

All laboratory-adapted *Toxoplasma* strains were maintained in HFF cells in high glucose Dulbecco's Modified Eagle Medium (DMEM) with sodium bicarbonate (Sigma) supplemented with 5% heat-inactivated fetal bovine serum (FBS) and 1% penicillin-streptomycin in humidified incubators at 37°C with 5% CO2 and ambient oxygen (21%). The Type II ME49EW strain was exclusively maintained in mice as described in reference (10). The ME49EW strain was continually passed through 5- to 6-week-old female mice by first infecting resistant strain SWR/J (Swiss Webster) with 10 cysts in 200 µL of cortex homogenate by i.p. injection [see reference (10)]. Tissue cysts in brain homogenates from infected SWR/J mice were then used to infect CBA/J, sensitive mice (10 cysts in 200 µL diluted brain homogenates, i.p.). The strain ME49EW1 was obtained by forced adaptation of the parent ME49EW strain to continuous growth in HFF cells under standard culture conditions and then cloned by limiting dilution. Genotype of the PruQ strain used in these studies is Type II Prugniaud Δ*ku80*, Δ*hxgprt::*LDH2-GFP (12). *Toxoplasma* parasites used for the infection of new host cells or animals and also used for the construction of scRNA-sequencing libraries were harvested and purified from host cell monolayers using standard *Toxoplasma* laboratory practices. In brief, parasites are liberated from infected host cells in flasks by scraping the monolayer, using needle passage to disrupt host cells, and then being filtered with a 3-µm filter to remove intact host cells. Parasites are then collected by centrifugation, resuspended in the appropriate media and enumerated on a hemocytometer.

#### Astrocyte purification

Neonatal C57Bl/6 mice born at 0–3 days were sacrificed via decapitation and brains (without cerebellum) were dissected and placed in cold wash media (DMEM and 2% fetal bovine serum). Brains were pooled and homogenized through a sterile 40 µm cell strainer (Corning) using a 3 mL syringe plunger. Homogenate was washed twice with cold wash media and spun at 2,000 rpm for 10 min at 4°C. Homogenate was resuspended with 6 mL per brain of prewarmed complete media [DMEM (Corning)]; 10% FBS; 1% Non-essential Amino Acids Mixture 100× (Biowhittaker Reagents Lonza), 1% GlutaMAX supplement 100× (Thermo Fisher Scientific), 1% penicillin-streptomycin (Genclone), 1% HEPES buffer solution 1M (Thermo Fisher Scientific). Resuspended cells were plated in non-vented T-25 cm^2^ flasks with loose caps at 37°C, 5% CO_2_. At days 3, 5, and 7, astrocyte media were replaced. To remove contaminating less adherent microglia and oligodendrocytes, astrocytes were shaken on days 8–10 at 260 rpm, 37°C for 1.5 h. Media were replaced and cultures were shaken for an additional 24 h at 100 rpm, 37°C. Astrocytes were lifted with 3 mL of 0.25% Trypsin EDTA, counted, and plated at a density of 1 × 10^6^ cells per T-75 cm^2^ vented flask until confluent.

### Animal experiments

All animal research was conducted in accordance with the Animal Welfare Act. C57Bl/6, CBA/J, and SWR/J #689 mice obtained from Jackson Laboratory (Jackson ImmunoResearch Laboratories, Inc., West Grove, PA, USA) were bred and maintained in a pathogen-free vivarium under the Institutional Animal Care and Use Committee protocols established at the University of California, Riverside, and the University of South Florida, Tampa. Complete protocols for brain tissue isolation, brain homogenate preparation, tissue cyst purification and quantification, and bradyzoite excystation can be obtained from a recent Biorxiv pre-print (10).

#### Infections of mice with laboratory strains and recrudescing populations

ME49EW bradyzoites and parasites at various times during recrudescence in HFF or astrocyte cells, as well as laboratory strains grown in HFF cells, were purified from host cell monolayers by standard scrape, needle pass, and filter methods and diluted to 50,000 parasites/mL in phosphate buffered saline (PBS). We have determined experimentally that filtration of needle-passed parasite cultures through 3 micron filters removes >95% of trypan blue positive (dead) parasites. Groups of CBA/J mice as indicated were infected i.p. with 0.2 mL of the appropriate inocula. Mouse infections were used to determine parasite burden, cytokine expression, and tissue cyst formation.

#### Serum cytokine ELISA

Serum was harvested from mice via tail vein bleeding on days 3, 5, 7, 9, and 14. Blood was centrifuged at 16,000 rpm for 20 min. Serum was aliquoted and stored at 80°C. IFNγ capture antibody (ebioscience: 14-7313-81) was incubated overnight at 4°C (1:1,000). Recombinant IFNγ (ebioscience, cat#: 14-8311-63) was used to create the standard. Standard and serum samples were incubated at 37°C for 2 h. Biotin-conjugated anti-mouse IFNγ (clone: R4-6A2; ebioscience: 13-7312-85) was incubated at 37°C for 1 h. Peroxidase conjugated streptavidin (Jackson immune research 016-030-084) was incubated for 30 min at 37°C. TMB substrate (Thermo, N301) is used for the colorimetric reaction, and an equal part of 0.16 M sulfuric acid is used to stop the reaction. Standard curve and analysis were conducted in GraphPad Prism (GraphPad Software).

#### Sulfamerazine treatment

CBA/j (25 mice total) mice were each inoculated with 10,000 excysted ME49EW bradyzoites by i.p. injection. At various times following infection (see Fig. 6 for details), sulfamerazine (444 mg/L) (43) was added to the drinking water of selected groups of mice. At 30 d.p.i., mice were euthanized, brain cortices removed, and tissue cysts in homogenates enumerated in triplicate.

### *Ex vivo* bradyzoite recrudescence experiments

Preliminary investigations determined that native ME49EW parasites grew better in HFF cells at lower oxygen levels (10). Therefore, throughout this study we cultured ME49EW parasites in HFF or primary astrocyte monolayers that were placed in a hypoxic chamber and grown in the same media with the exception that astrocytes received a 1% GlutaMAX supplement [for full methods, see reference (10)]. Confluent HFF or astrocyte monolayers were inoculated at various times with excysted bradyzoites, lab strains, or recrudescing populations at an MOI of ~0.5. ME49EW parasites were passaged into new monolayers of either host cell type using the schedules shown in Fig. S1A. To determine population growth changes, HFF or astrocyte monolayers were infected (MOI 1:1) and harvested using the schedule described in Fig. S1A. Parasites were harvested using the protocol listed above (parasite strains and host cells) and parasite numbers at each time point were enumerated on a hemocytometer. Changes in parasite population growth over 7 days (Day-3, Day-5, and Day-7) in HFF or astrocytes in comparison to the number of parasites used for infection were independently measured three times. The population measurements are estimates of growth that assume 100% infection.

#### Growth rate doublings

Post-excysted bradyzoites and Day 3- and Day 5-recrudesing populations were inoculated onto 6-well coverslips of HFF or astrocyte cells and, at various times, were fixed and stained using an anti-toxoplasma antibody (Abcam, ab138698). Vacuole sizes at each timepoint were determined by counting 50 random vacuoles in triplicate on a Zeiss Axiovert fluorescent microscope. For each coverslip, the average number of parasite doublings per day was determined from the raw vacuole sizes (vacuole of 2 = 1 doubling, vacuole of 4 = 2 doublings, vacuole of 8 = 3 doublings, etc.) divided by the total days in each incubation period.

#### Visualization of parasite growth and development

ME49EW bradyzoites and various recrudescing populations were inoculated onto six-well glass coverslips of HFF or astrocyte cells at 0.5 MOI. All coverslips were cultured under low oxygen conditions as described [see reference (10)]. Infected coverslips were fixed with 4% paraformaldehyde (PFA) for 10 min. Cells were permeabilized with either 100% acetone or 0.25% Triton-X for 10 min. Cells were blocked with either 5% donkey serum or 1% bovine serum albumin (BSA) for 30 min, followed by 1-h incubation of the following primary antibodies diluted in blocking buffer: biotinylated-DBA (1:500) (Vector Laboratories), rabbit-anti SRS9 (1:1,000), and mouse-anti SAG1 (1:1,000) (kindly provided by John Boothroyd, Stanford University), rabbit-anti-Toxo (1:500) (Abcam), mouse-anti-CST-1 (1:1,000) (kindly provided by Louis Weiss, Albert Einstein College of Medicine), mouse-anti-IMC1 (1:1,000) (kindly provided by Gary Ward, University of Vermont), rabbit-anti-centrin (1:500) (used 2-h primary incubation). Secondary antibody master mix was incubated for 1 h: Goat-anti-rabbit-AF568 (1:1,000), Goat-anti-mouse-AF488 (1:1,000), streptavidin-AF647 (1:1,000), and DAPI (1 mg/mL). All incubations were done at room temperature, and all washes used 1× PBS.

### Cell preparation and flow cytometry

#### Cell preparation

PECS and blood were collected from naïve and infected mice at each time point using injection and reuptake of 1× PBS using a 22G needle and 5 mL syringe (for PECS). Volume collected was recorded. Blood was collected by cutting right atrium of the heart with scissors and collecting approximately 500 µL. Blood was subjected to two rounds of ammonium-chloride-potassium (ACK) Lysis Buffer (500 µL) and pipetted through the filter cap to minimize debris. PECS and blood cells were counted using an automated cell counter and 1.0 × 10^6^ live cells were taken for flow cytometry staining.

#### Flow cytometry

During cell preparation and flow cytometry staining, all spins were conducted at 3,000 rpm for 5 min at 4°C to spin down extracellular parasites as well as infected cells. All cells were incubated in FC Block (BD Pharmingen: 553142) to minimize non-specific antibody binding, incubated with CD45 antibody conjugated to phycoerythrin (PE) (eBioscience: Clone 30-F11) (1:100) to identify immune cells, fixed with 4% PFA (15 min at room temperature), and permeabilized via 0.3% saponin spin for intracellular staining. Cells were then incubated with primary Rabbit anti-Toxo antibody (Abcam, ab138698) (1:200) followed by Goat anti-rabbit AlexaFluor488 secondary stain (eBioscience) (1:500) to identify both intracellular and extracellular parasites. All intracellular stain steps were completed in 0.3% saponin to maintain permeabilization. In between antibody incubations, cells were spun down to discard any unbound antibody. Upon completion of intracellular staining, cells were spun in fluorescent activated cell sorting (FACS) buffer to close the membrane, and cells were resuspended in 300 µL FACS buffer for flow cytometry. Flow cytometry was run on BD FACSCanto II, and analysis was performed using FlowJo software.

### Parasite burden

Organ tissues (brain, lung, muscle, liver, and heart) and PECS were harvested from mice and stored at −80°C prior to DNA purification. DNA was purified using a DNeasy minikit (Qiagen) according to the manufacturer’s protocols. The qPCR reaction volume used 600 ng of DNA from each organ, 0.375 µM B1 gene primers (forward: 5′-TCCCCTCTGCTGGCGAAAAGT-3′; reverse: 5′-AGCGTTCGTGGTCAACTATCG-3′), and 2× Luna universal qPCR master mix (New England Biolabs) in a 25-µL total volume. The amplification program was 3 min at 95°C, 40 cycles of 10 s at 95°C for denaturation, 30 s at 60°C for annealing, followed by 5 s at 65°C and 5 s at 95°C for extension. The real-time data were collected on a CFX96 Real-Time system, C1000 Touch thermal cycler using Bio-Rad CFX Maestro software. A standard curve was generated based on the serial dilution of purified ME49 genomic DNA. All results were expressed as the pg of parasite genomic DNA calculated from an experimentally generated standard curve generated in each PCR run.

### Gene expression

#### Total RNA-sequencing analyses

Total RNA from ME49 cysts and tachyzoites and alkaline-induced ME49 bradyzoites (pH 8.2 media for 72 h) was purified using the RNeasay kit (Qiagen) according to the manufacturer’s protocols. RNA quality was assessed using an Agilent 2100 Bioanalyzer (Waldbronn, Germany). RNA samples were processed for RNA sequencing using the NuGen Universal RNA-Sequencing System (NuGEN Technologies, San Carlos, CA). Briefly, 100 ng of RNA was used to generate cDNA and a strand-specific library following the manufacturer’s protocol. In order to deplete contaminating abundant human and *T. gondii* rRNA sequences, a cocktail of NuGEN human AnyDeplete rRNA-targeting probes and a custom panel of 196T. *gondii* AnyDeplete probes were used to carry out insert-dependent adaptor cleavage, as described in the manufacturer’s protocol. The final cDNA libraries were evaluated on the BioAnalyzer and quantified using the Kapa Library Quantification Kit (Roche Sequencing, Pleasanton, CA) by the Molecular Genomics Core Facility (Moffitt Cancer Center, FL). The libraries were then sequenced on the Illumina NextSeq 500 sequencer with 75-base paired-end runs in order to generate at least 10 million read pairs per sample. Reads were filtered by Sanger quality score using FASTQ Groomer v. 1.0.4 and paired-end reads were aligned against the genomes of *T. gondii* (TGME49 version 46; ToxoDB.org) references uploaded into Galaxy using HISAT2. The DESeq2 module was used for normalization, differential gene expression, and statistical analysis of uniquely mapped paired-end reads using the default parameters. DEseq2 is a bioconductor package that is freely available for analysis of total RNA-sequencing data (https://bioconductor.org/packages/release/bioc/html/DESeq2.html). DEseq2 estimates the variance-mean dependence in mRNA count data from high-throughput sequencing assays and tests for differential expression based on a statistical model using a negative binomial distribution method (*P*-value ≤ 0.05). Changes in mRNA expression in the *in vivo* bradyzoite were filtered using a log2 cutoff of >1 and <−1 (±twofold). Cell cycle genes showing a cyclical expression pattern were identified rigorously by two methods: first, a criterion of significantly elevated variance was applied (ANOVA FDR = 0.1); second, a cubic B-spline criterion with empirical confidence intervals determined from 1,000 simulations was applied to the synchronous time course [see reference (16) for full analysis details].

#### Single-cell RNA sequencing

Single-cell RNA sequencing was performed on parasites from respective conditions (see Fig. 3 and 4; Fig. S2 for examples) using the 10× Genomics Chromium Next GEM Single Cell 3ʹ Reagent Kits v3.1 as described in the manufacturer’s protocol. Sequencing depth was performed at 1 billion reads and generated at the UC San Diego IGM Genomics Center utilizing an Illumina NovaSeq 6000 that was purchased with funding from a National Institutes of Health SIG grant (#S10 OD026929). Single-cell RNA seq data were analyzed using Cell Ranger (version 5.0.1), which consists of a series of pipelines that are capable of alignment to a reference genome, generating unique molecular identifier (UMI) counts, and clustering. The sequencing reads were aligned to the ME49 *T. gondii* reference genome. Clustering and differential gene expression analysis were performed in the cLoupe browser (v6.4.1). Statistically significant differences in gene expression were identified using an exact negative binomial test (44) that incorporates size factors calculated using total gene-specific UMI counts rather than geometric means. Cell Ranger uses a mutual nearest neighbors algorithm to identify different clusters of cells (45) that can be visualized by graph-based clustering or K-means clustering. Some QC metrics, such as the violin plots (Fig. S3B) were generated using R version 4.2.3 with the Seurat package (version 4.3.0). Based on the violin plots, a 500 UMI cutoff was applied to all samples and those cells that were below this cutoff were filtered out. The remaining cells were used for K-means clustering analysis on cLoupe Browser. Further information can be found on the 10× genomics website: https://support.10xgenomics.com/single-cell-gene-expression/software/downloads/latest

### Cell cycle analyses

#### Genome DNA content

Excysted bradyzoites and freshly lysed and purified RH parasites were pelleted at 2,000 rpm for 15 min at 4°C and resuspended in 300 µL of cold 70% ethanol in PBS added dropwise. Samples were held at −20°C at least 24 h prior to staining. Samples were pelleted by centrifuging at 6,500 rpm for 5 min; the pellet was then resuspended in staining solution [50 µM Tris pH 7.5, 1 µM SYTOX green (Molecular Probes #S7020) and 1% RNase cocktail] and incubated for 30 min at room temperature in the dark. Flow cytometry analysis was performed using a BD-Canto flow cytometer. The cytometer was run in linear mode, calibrated to the 1N population of the RH parasite reference control, and 70,000 events were collected per sample. Histograms were generated using FACSDiva software.

#### Cell cycle IFA

Excysted bradyzoites were cytospun (500 rpm for 5 min medium acceleration) onto slides, then co-stained for IMC1 and genomic DNA (DAPI) or separately co-stained for SRS9 and SAG1. IFA analysis of ME49EW Day 2-, Day 7-, and Day 9-recrudescent populations was also performed by co-staining with antibodies against IMC1 and centrin and DAPI (genomic DNA). Parasite vacuoles were quantified for single (G1 phase) vs. double (S/M phases) centrosomes, and for the presence of internal daughters in 50 randomly selected vacuoles ×3 for each sample.

## Data Availability

All new transcriptome data included in this study can be obtained from GEO: total RNA sequencing, accession no. GSE208647; scRNA sequencing, accession no. GSE210671.

## SUPPLEMENTAL MATERIAL

Database S1 mbio.01836-23-s0001.xlsx Total RNA-sequencing.

Database S2 mbio.01836-23-s0002.xlsx Single cell RNA-sequencing.

Figure S1 mbio.01836-23-s0003.tif Growth and antigen expression supplemental data for excysted ME49EW bradyzoites and recrudescent parasites.

Figure S2 mbio.01836-23-s0004.tif Changes in developmental antigen expression in HFF cells.

Figure S3 mbio.01836-23-s0005.tif scRNA-seq QA.

Figure S4 mbio.01836-23-s0006.tif scRNA-seq supplemental data.

Figure S5 mbio.01836-23-s0007.tif Supplemental results for Figures 6 and 7.

Supplemental Legends mbio.01836-23-s0008.pdf Legends for supplemental figures and databases.

ASM does not own the copyrights to Supplemental Material that may be linked to, or accessed through, an article. The authors have granted ASM a non-exclusive, world-wide license to publish the Supplemental Material files. Please contact the corresponding author directly for reuse.

## References

[1] Dubey JP . 1997. Tissue cyst tropism in Toxoplasma gondii: a comparison of tissue cyst formation in organs of cats, and rodents fed oocysts. Parasitology 115 ( Pt 1):15–20. doi:10.1017/s0031182097008949 9226953

[2] Dubey JP , Frenkel JK . 1976. Feline toxoplasmosis from acutely infected mice and the development of Toxoplasma cysts. J Protozool 23:537–546. doi:10.1111/j.1550-7408.1976.tb03836.x 1003342

[3] Weiss LM , Dubey JP . 2009. Toxoplasmosis: a history of clinical observations. Int J Parasitol 39:895–901. doi:10.1016/j.ijpara.2009.02.004 19217908 PMC2704023

[4] Remington JS . 1974. Toxoplasmosis in the adult. Bull N Y Acad Med 50:211–227. doi:10.1056/NEJM197310252891702 4592097 PMC1749356

[5] Song HB , Jung B-K , Kim JH , Lee Y-H , Choi M-H , Kim JH . 2018. Investigation of tissue cysts in the retina in a mouse model of ocular toxoplasmosis: distribution and interaction with glial cells. Parasitol Res 117:2597–2605. doi:10.1007/s00436-018-5950-3 29858945

[6] Dellacasa-Lindberg I , Hitziger N , Barragan A . 2007. Localized recrudescence of Toxoplasma infections in the central nervous system of immunocompromised mice assessed by in vivo bioluminescence imaging. Microbes Infect 9:1291–1298. doi:10.1016/j.micinf.2007.06.003 17897859

[7] Odaert H , Soête M , Fortier B , Camus D , Dubremetz JF . 1996. Stage conversion of Toxoplasma gondii in mouse brain during infection and immunodepression. Parasitol Res 82:28–31. doi:10.1007/BF03035408 8825441

[8] Reiter-Owona I , Seitz H , Gross U , Sahm M , Rockstroh JK , Seitz HM . 2000. Is stage conversion the initiating event for reactivation of Toxoplasma gondii in brain tissue of AIDS patients? J Parasitol 86:531–536. doi:10.1645/0022-3395(2000)086[0531:ISCTIE]2.0.CO;2 10864251

[9] Howe DK , Honoré S , Derouin F , Sibley LD . 1997. Determination of genotypes of Toxoplasma gondii strains isolated from patients with toxoplasmosis. J Clin Microbiol 35:1411–1414. doi:10.1128/jcm.35.6.1411-1414.1997 9163454 PMC229759

[10] Hong DD , Brooks K , Goerner AL , Vizcarra EA , Loges LN , Wilson EH , White MW . 2022. Engineering toxoplasma transgenic tissue cysts. Biorxiv pre-print server. doi:10.1101/2022.07.21.500998

[11] Saraf P , Shwab EK , Dubey JP , Su C . 2017. On the determination of Toxoplasma gondii virulence in mice. Exp Parasitol 174:25–30. doi:10.1016/j.exppara.2017.01.009 28153801

[12] Fox BA , Falla A , Rommereim LM , Tomita T , Gigley JP , Mercier C , Cesbron-Delauw M-F , Weiss LM , Bzik DJ . 2011. Type II Toxoplasma gondii KU80 knockout strains enable functional analysis of genes required for cyst development and latent infection. Eukaryot Cell 10:1193–1206. doi:10.1128/EC.00297-10 21531875 PMC3187049

[13] Radke JR , Guerini MN , Jerome M , White MW . 2003. A change in the premitotic period of the cell cycle is associated with bradyzoite differentiation in Toxoplasma gondii. Mol Biochem Parasitol 131:119–127. doi:10.1016/s0166-6851(03)00198-1 14511810

[14] Olguin-Lamas A , Madec E , Hovasse A , Werkmeister E , Callebaut I , Slomianny C , Delhaye S , Mouveaux T , Schaeffer-Reiss C , Van Dorsselaer A , Tomavo S . 2011. A novel Toxoplasma gondii nuclear factor TgNF3 is a dynamic chromatin-associated component, modulator of nucleolar architecture and parasite virulence. PLoS Pathog 7:e1001328. doi:10.1371/journal.ppat.1001328 21483487 PMC3068996

[15] Radke JR , Behnke MS , Mackey AJ , Radke JB , Roos DS , White MW . 2005. The transcriptome of Toxoplasma gondii. BMC Biol 3:26. doi:10.1186/1741-7007-3-26 16324218 PMC1325263

[16] Behnke MS , Wootton JC , Lehmann MM , Radke JB , Lucas O , Nawas J , Sibley LD , White MW . 2010. Coordinated progression through two subtranscriptomes underlies the tachyzoite cycle of Toxoplasma gondii. PLoS One 5:e12354. doi:10.1371/journal.pone.0012354 20865045 PMC2928733

[17] Radke JR , Striepen B , Guerini MN , Jerome ME , Roos DS , White MW . 2001. Defining the cell cycle for the tachyzoite stage of Toxoplasma gondii. Mol Biochem Parasitol 115:165–175. doi:10.1016/s0166-6851(01)00284-5 11420103

[18] Schlüter D , Barragan A . 2019. Advances and challenges in understanding cerebral toxoplasmosis. Front Immunol 10:242. doi:10.3389/fimmu.2019.00242 30873157 PMC6401564

[19] Cabral CM , Tuladhar S , Dietrich HK , Nguyen E , MacDonald WR , Trivedi T , Devineni A , Koshy AA . 2016. Neurons are the primary target cell for the brain-tropic intracellular parasite Toxoplasma gondii. PLoS Pathog 12:e1005447. doi:10.1371/journal.ppat.1005447 26895155 PMC4760770

[20] Jerome ME , Radke JR , Bohne W , Roos DS , White MW . 1998. Toxoplasma gondii bradyzoites form spontaneously during sporozoite-initiated development. Infect Immun 66:4838–4844. doi:10.1128/IAI.66.10.4838-4844.1998 9746587 PMC108598

[21] Gaji RY , Behnke MS , Lehmann MM , White MW , Carruthers VB . 2011. Cell cycle-dependent, intercellular transmission of Toxoplasma gondii is accompanied by marked changes in parasite gene expression. Mol Microbiol 79:192–204. doi:10.1111/j.1365-2958.2010.07441.x 21166903 PMC3075969

[22] Ovciarikova J , Lemgruber L , Stilger KL , Sullivan WJ , Sheiner L . 2017. Mitochondrial behaviour throughout the lytic cycle of Toxoplasma gondii. Sci Rep 7:42746. doi:10.1038/srep42746 28202940 PMC5311943

[23] Baggish AL , Hill DR . 2002. Antiparasitic agent atovaquone. Antimicrob Agents Chemother 46:1163–1173. doi:10.1128/AAC.46.5.1163-1173.2002 11959541 PMC127192

[24] Thapa M , Bommakanti A , Shamsuzzaman M , Gregory B , Samsel L , Zengel JM , Lindahl L . 2013. Repressed synthesis of ribosomal proteins generates protein-specific cell cycle and morphological phenotypes. Mol Biol Cell 24:3620–3633. doi:10.1091/mbc.E13-02-0097 24109599 PMC3842990

[25] Mordue DG , Monroy F , La Regina M , Dinarello CA , Sibley LD . 2001. Acute toxoplasmosis leads to lethal overproduction of Th1 cytokines. J Immunol 167:4574–4584. doi:10.4049/jimmunol.167.8.4574 11591786

[26] Konradt C , Ueno N , Christian DA , Delong JH , Pritchard GH , Herz J , Bzik DJ , Koshy AA , McGavern DB , Lodoen MB , Hunter CA . 2016. Endothelial cells are a replicative niche for entry of Toxoplasma gondii to the central nervous system. Nat Microbiol 1:16001. doi:10.1038/nmicrobiol.2016.1 27572166 PMC4966557

[27] Watson GF , Davis PH . 2019. Systematic review and meta-analysis of variation in Toxoplasma gondii cyst burden in the murine model. Exp Parasitol 196:55–62. doi:10.1016/j.exppara.2018.12.003 30562481 PMC6447088

[28] Radke JB , Worth D , Hong D , Huang S , Sullivan WJ , Wilson EH , White MW . 2018. Transcriptional repression by ApiAP2 factors is central to chronic toxoplasmosis. PLoS Pathog 14:e1007035. doi:10.1371/journal.ppat.1007035 29718996 PMC5951591

[29] Lindsay DS , Dubey JP , Blagburn BL , Toivio-Kinnucan M . 1991. Examination of tissue cyst formation by Toxoplasma gondii in cell cultures using bradyzoites, tachyzoites, and sporozoites. J Parasitol 77:126–132. 1992083

[30] Shukla A , Olszewski KL , Llinás M , Rommereim LM , Fox BA , Bzik DJ , Xia D , Wastling J , Beiting D , Roos DS , Shanmugam D . 2018. Glycolysis is important for optimal asexual growth and formation of mature tissue cysts by Toxoplasma gondii. Int J Parasitol 48:955–968. doi:10.1016/j.ijpara.2018.05.013 30176233

[31] Ferguson DJ , Hutchison WM . 1987. An ultrastructural study of the early development and tissue cyst formation of Toxoplasma gondii in the brains of mice. Parasitol Res 73:483–491. doi:10.1007/BF00535321 3422976

[32] Watts E , Zhao Y , Dhara A , Eller B , Patwardhan A , Sinai AP . 2015. Novel approaches reveal that Toxoplasma gondii bradyzoites within tissue cysts are dynamic and replicating entities in vivo. mBio 6:e01155–15. doi:10.1128/mBio.01155-15 26350965 PMC4600105

[33] Denton H , Roberts CW , Alexander J , Thong KW , Coombs GH . 1996. Enzymes of energy metabolism in the bradyzoites and tachyzoites of Toxoplasma gondii. FEMS Microbiol Lett 137:103–108. doi:10.1111/j.1574-6968.1996.tb08090.x 8935663

[34] Cerutti A , Blanchard N , Besteiro S . 2020. The bradyzoite: a key developmental stage for the persistence and pathogenesis of toxoplasmosis. Pathogens 9:234. doi:10.3390/pathogens9030234 32245165 PMC7157559

[35] Dubey JP . 2005. Unexpected oocyst shedding by cats fed Toxoplasma gondii tachyzoites: in vivo stage conversion and strain variation. Vet Parasitol 133:289–298. doi:10.1016/j.vetpar.2005.06.007 16024176

[36] Dzierszinski F , Nishi M , Ouko L , Roos DS . 2004. Dynamics of Toxoplasma gondii differentiation. Eukaryot Cell 3:992–1003. doi:10.1128/EC.3.4.992-1003.2004 15302832 PMC500887

[37] Tanaka N , Ashour D , Dratz E , Halonen S . 2016. Use of human induced pluripotent stem cell-derived neurons as a model for cerebral toxoplasmosis. Microbes Infect 18:496–504. doi:10.1016/j.micinf.2016.03.012 27083472

[38] Halonen SK , Taylor GA , Weiss LM . 2001. Gamma interferon-induced inhibition of Toxoplasma gondii in astrocytes is mediated by IGTP. Infect Immun 69:5573–5576. doi:10.1128/IAI.69.9.5573-5576.2001 11500431 PMC98671

[39] Hidano S , Randall LM , Dawson L , Dietrich HK , Konradt C , Klover PJ , John B , Harris TH , Fang Q , Turek B , Kobayashi T , Hennighausen L , Beiting DP , Koshy AA , Hunter CA . 2016. Stat1 signaling in astrocytes is essential for control of infection in the central nervous system. mBio 7:e01881-16. doi:10.1128/mBio.01881-16 27834206 PMC5101356

[40] Rani S , Cerqueira-Cézar CK , Murata FHA , Sadler M , Kwok OCH , Pradhan AK , Hill DE , Urban JF , Dubey JP . 2019. Toxoplasma gondii tissue cyst formation and density of tissue cysts in shoulders of pigs 7 and 14 days after feeding infected mice tissues. Vet Parasitol 269:13–15. doi:10.1016/j.vetpar.2019.04.004 31079821

[41] Ten Hoeve AL , Hakimi M-A , Barragan A . 2019. Sustained Egr-1 response via P38 MAP kinase signaling modulates early immune responses of dendritic cells parasitized by Toxoplasma gondii. Front Cell Infect Microbiol 9:349. doi:10.3389/fcimb.2019.00349 31681626 PMC6797980

[42] Xue Y , Theisen TC , Rastogi S , Ferrel A , Quake SR , Boothroyd JC . 2020. A single-parasite transcriptional atlas of Toxoplasma gondii reveals novel control of antigen expression. Elife 9:e54129. doi:10.7554/eLife.54129 32065584 PMC7180058

[43] Buchholz KR , Fritz HM , Chen X , Durbin-Johnson B , Rocke DM , Ferguson DJ , Conrad PA , Boothroyd JC . 2011. Identification of tissue cyst wall components by transcriptome analysis of in vivo and in vitro Toxoplasma gondii bradyzoites. Eukaryot Cell 10:1637–1647. doi:10.1128/EC.05182-11 22021236 PMC3232729

[44] Yu D , Huber W , Vitek O . 2013. Shrinkage estimation of dispersion in negative binomial models for RNA-Seq experiments with small sample size. Bioinformatics 29:1275–1282. doi:10.1093/bioinformatics/btt143 23589650 PMC3654711

[45] Haghverdi L , Lun ATL , Morgan MD , Marioni JC . 2018. Batch effects in single-cell RNA-sequencing data are corrected by matching mutual nearest neighbors. Nat Biotechnol 36:421–427. doi:10.1038/nbt.4091 29608177 PMC6152897

