# Supplementary figures and images for: An ex vivo model of Toxoplasma recrudescence reveals developmental plasticity of the bradyzoite stage
Source: mBio. 2023 Sep 7;14(5):e01836-23. doi: 10.1128/mbio.01836-23 (PMC10653814; doi:10.1128/mbio.01836-23)

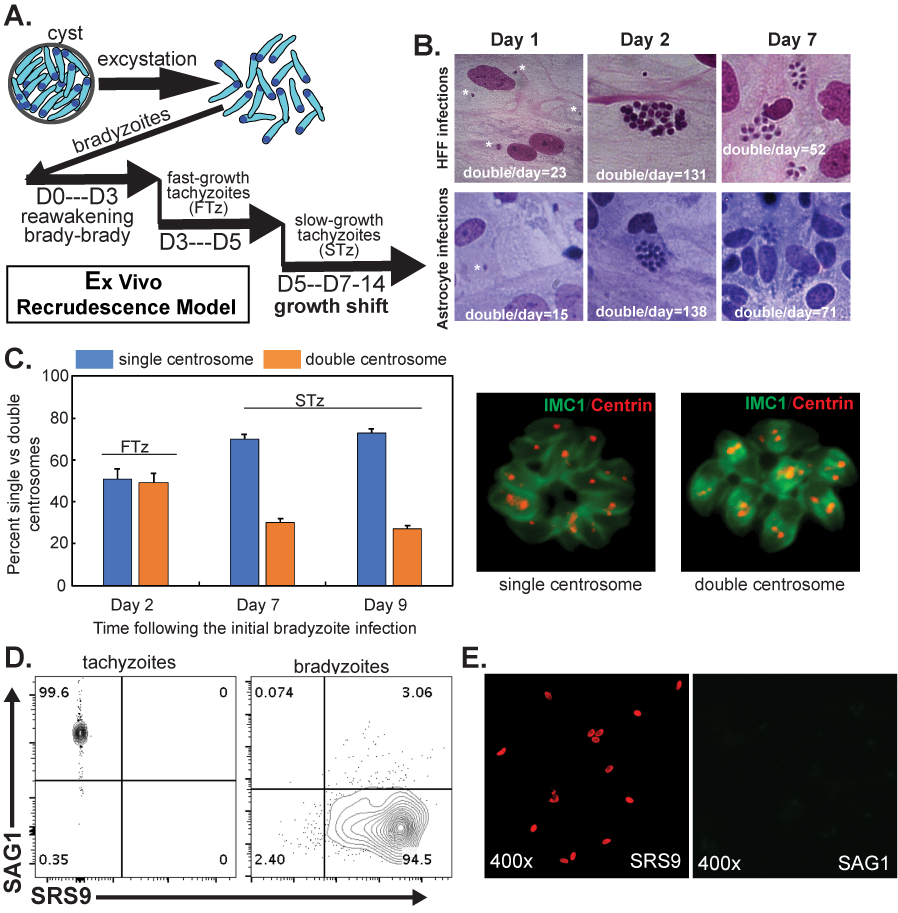

Supplement: Figure S1 — Growth and antigen expression supplemental data for excysted ME49EW bradyzoites and recrudescent parasites. [file mbio.01836-23-s0003.tif]

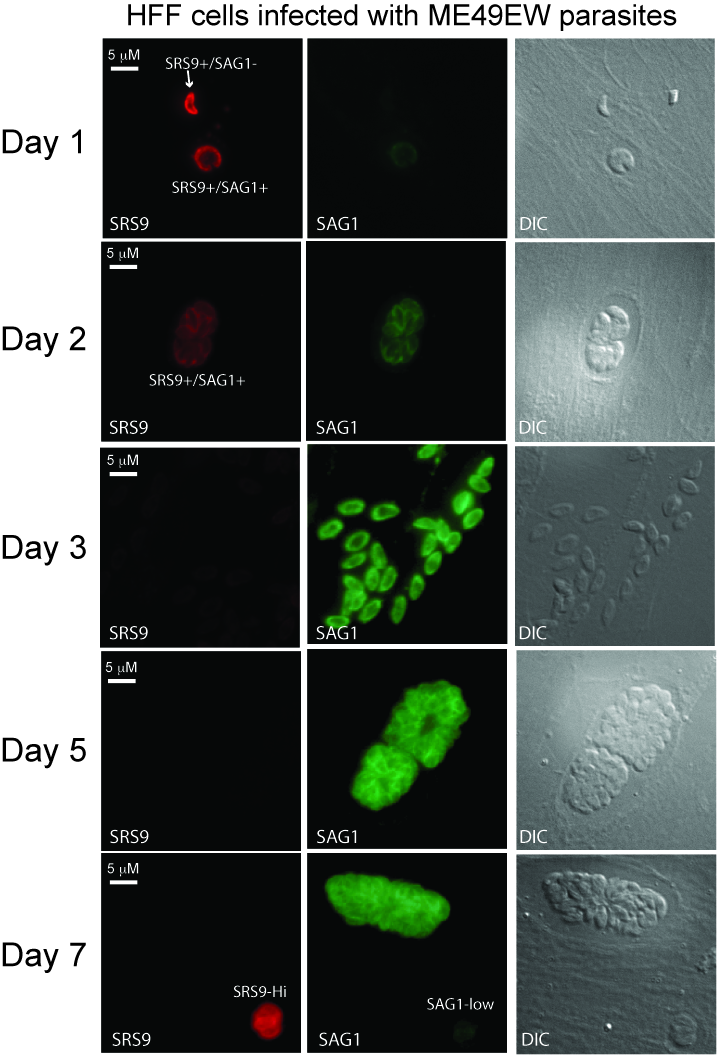

Supplement: Figure S2 — Changes in developmental antigen expression in HFF cells. [file mbio.01836-23-s0004.tif]

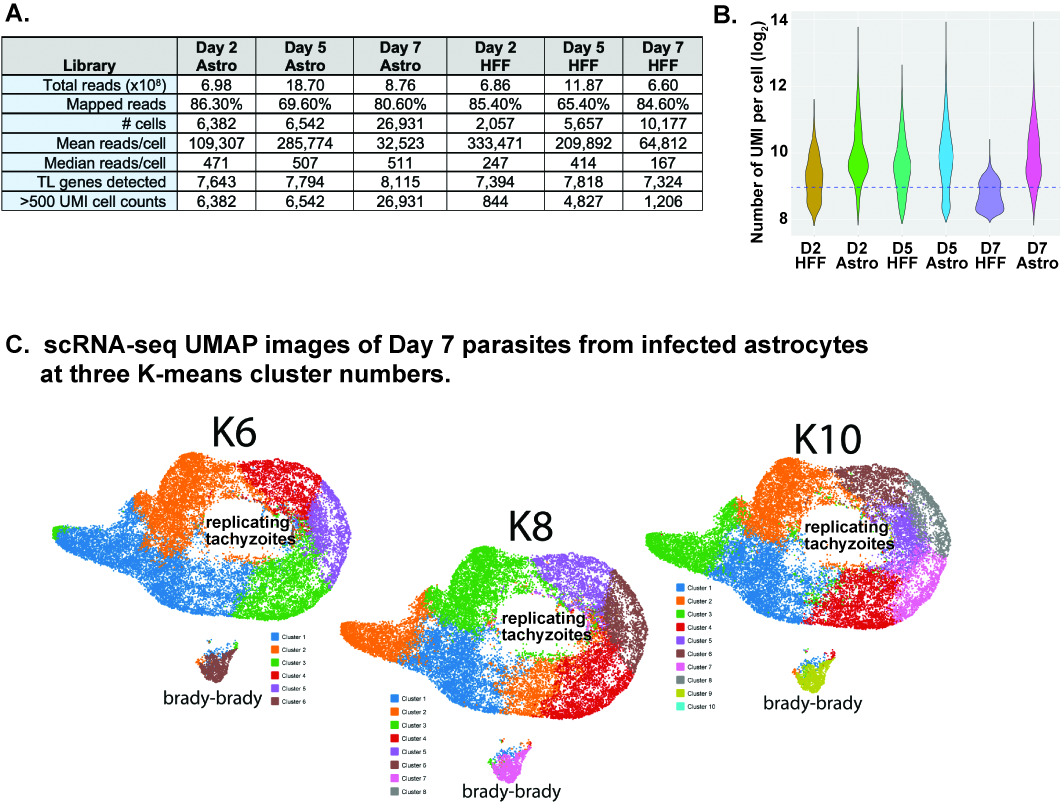

Supplement: Figure S3 — scRNA-seq QA. [file mbio.01836-23-s0005.tif]

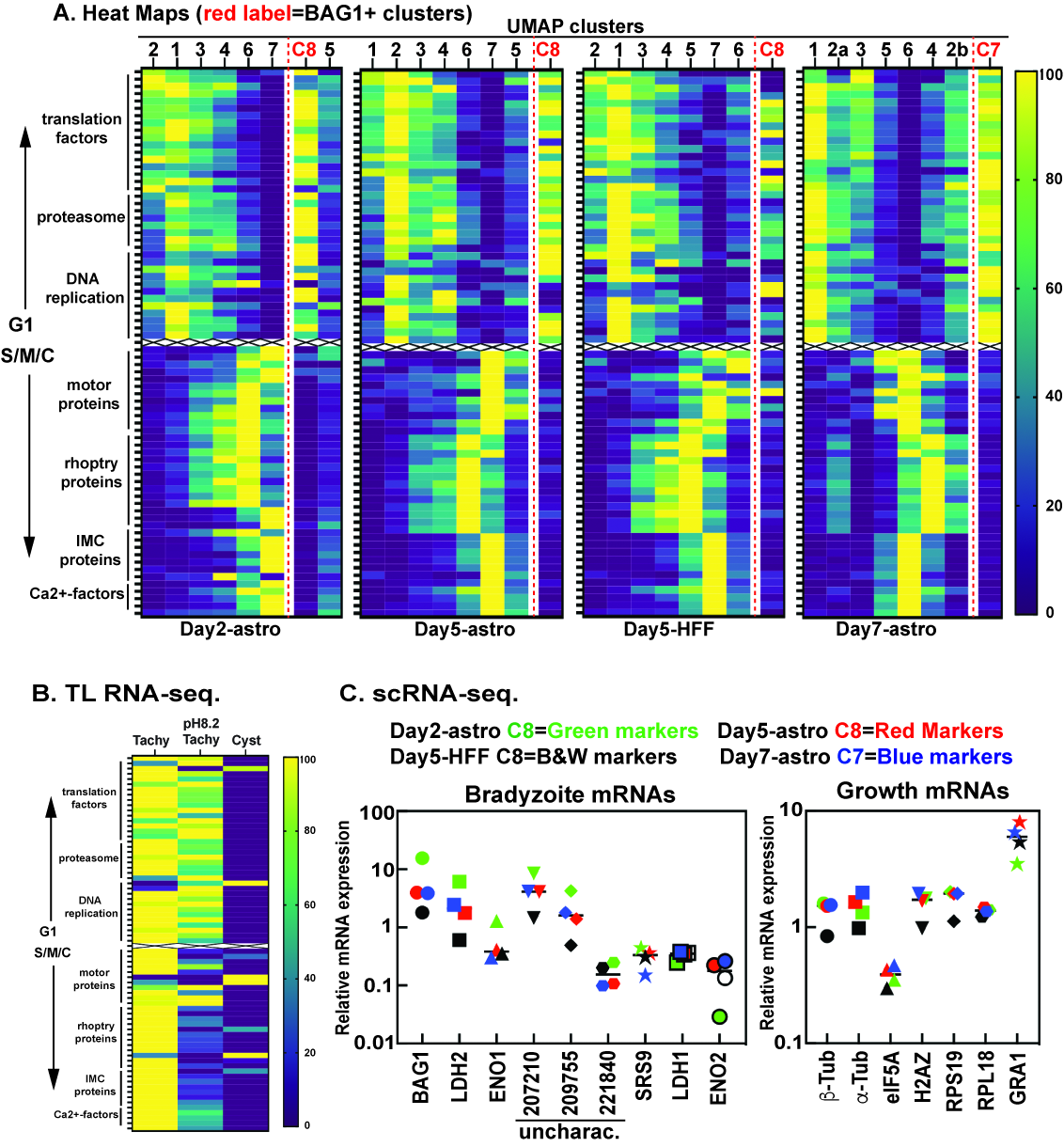

Supplement: Figure S4 — scRNA-seq supplemental data. [file mbio.01836-23-s0006.tif]

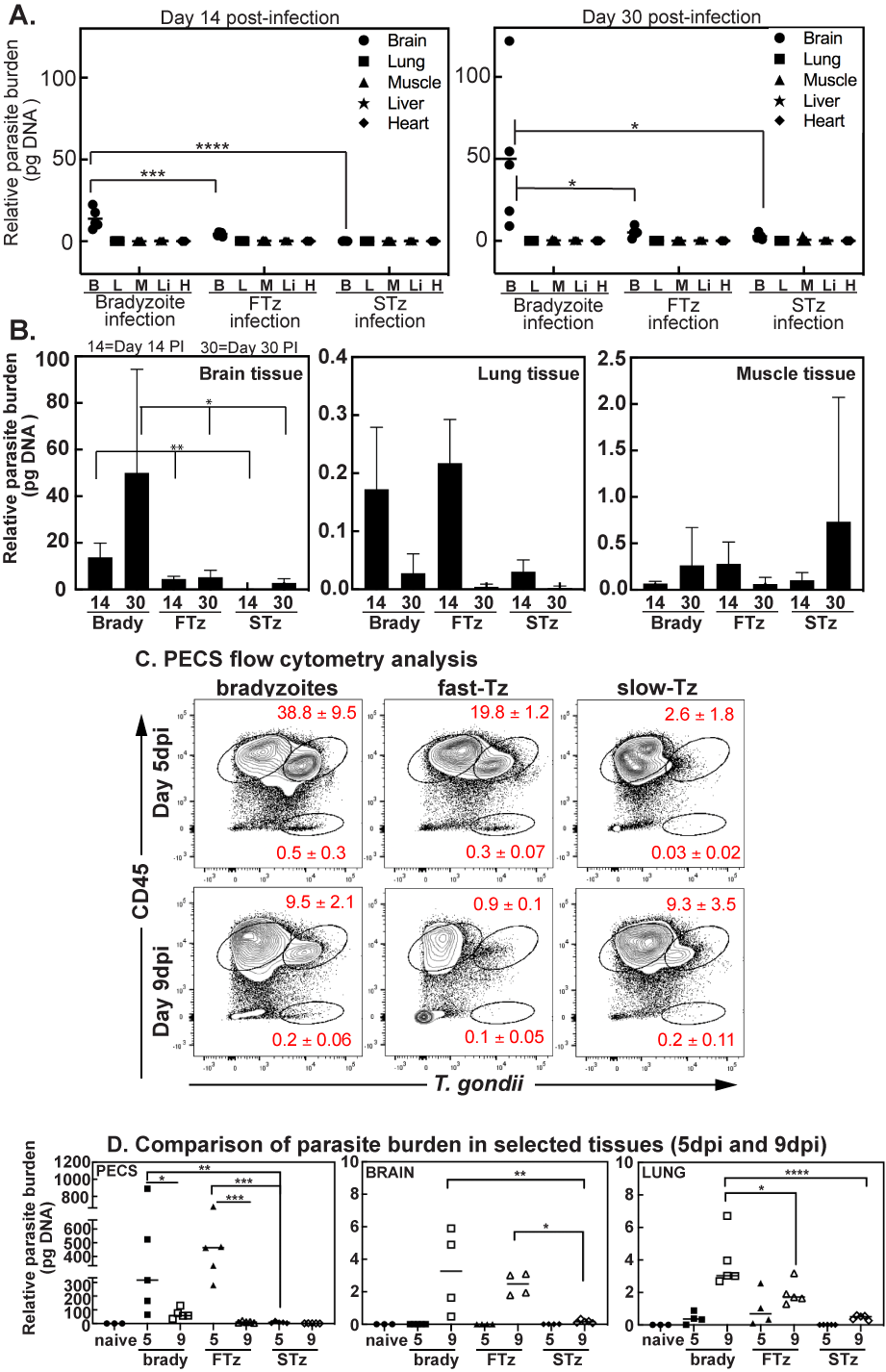

Supplement: Figure S5 — Supplemental results for Figures 6 and 7. [file mbio.01836-23-s0007.tif]
