# Supplementary material for: An ex vivo model of Toxoplasma recrudescence reveals developmental plasticity of the bradyzoite stage
Source: mBio. 2023 Sep 7;14(5):e01836-23. doi: 10.1128/mbio.01836-23 (PMC10653814; doi:10.1128/mbio.01836-23)
Supplement: Supplemental Legends — Legends for supplemental figures and databases. [file mbio.01836-23-s0008.pdf]

Supplement Figure Legends

Figure S1. Growth and antigen expression supplemental data for excysted ME49EW bradyzoites and recrudescent parasites.

[A.] Diagram: parasite populations were followed for 1-2 weeks with timepoints labeled by accumulated time from the original bradyzoite infection (D0). Day-3 populations (fast-growing=FTz) are 72 h host cell cultures infected with bradyzoites; Day-5 populations are 48 h cultures infected with Day-3 parasites; Day-7 populations (slow-growing=STz) are 48 h cultures infected with Day-5 parasites etc. **[B.]** At various times, H&E stains (nuclei stain dark blue to violet) were prepared. Representative images of the major vacuole sizes are shown. Total doublings (e.g., vacuole of 32=5 doublings) indicated at the bottom of each image indicate relative growth rates (see Material and Methods). Day-2 doublings, which represent growth from the end of Day 1 to the end of Day 2, is a statistically significant increase in growth rate ($P<0.001$) . Asterisks in the top two images indicate the single bradyzoite vacuoles. **[C.]** At Day 2 (first monolayer), 7 (3rd monolayer), and 9 (4th monolayer) from the initial bradyzoite infection, parasites were fixed and co-stained for anti-centrin (centrosome), Dapi (DNA), and anti-IMC1 (internal daughters). Single versus double centrosomes were quantified in 3 x 50 vacuoles selected at random. Statistically significant difference in Day-2 vs Day-7or -9 single centrosome levels was observed ($P=0.02$). The merged images (IMC1 and Centrin co-staining) to the right are representative of parasites possessing single versus double centrosomes. Note the lengthening of G1 (increased single centrosome fraction) accounts for the longer division times of parasites from Day-7 and -9 infections **[D.]** Samples of RH tachyzoites and ME49EW bradyzoites were labeled with SRS9 and SAG1 antibodies and analyzed by flow cytometry. **[E.]** Excysted bradyzoites from in vivo tissue cysts were co-stained for SRS9 (bradyzoite stain) and SAG1 (tachyzoite stain).

Figure S2. Changes in developmental antigen expression in HFF cells.

Representative images of parasite staining patterns Day 1-3 post-infection of HFF cells infected with in vivo ME49EW bradyzoites purified from mouse brain. Day-5 and Day-7 images are of HFF cells infected with parasites purified from Day-3 or Day-5 parasite populations and then grown for 48 h to yield Day-5 images and Day-7 images, respectively. Parasites were fixed and co-stained for SAG1 (green), SRS9 (red). Paired DIC images are shown on the right.

Figure S3. scRNA-seq QA.

[A. and B.] scRNA-seq library statistics and the distribution of UMIs detected in the parasites from each library sample. Dashed line in [B.] indicates the minimum 500 UMI quality control cutoff aeqpplied to the parasites in each sample. Parasites from HFF cells showed comparatively lower quality than from astrocytes. **[C.]** An example of varying K number on UMAP clustering profiles; scRNA-seq data from Day-7 parasites from astrocytes was analyzed using three clustering levels; K6, K8, K10. Note all three UMAP profiles are similar indicating that replicating tachyzoite populations were well resolved from replicating bradyzoites at all three clustering levels. The resolution of tachyzoite cell cycle populations was optimal at the K8 level (Fig. S4A), therefore K8 clustering was chosen for the analysis of all scRNA-seq datasets.

Figure S4 scRNA-seq supplemental data.

[A.] Normalized mRNA expression of selected G1 and S/M/C cell cycle mRNAs (see Database S1 for CC gene list) are presented in four heat maps (scRNA-seq samples: Day-2, -5, -7 infected astrocytes and infected Day-5 HFF cells). Unsupervised clustering of scRNA-seq data yielded parasite groups (BAG1-, tachyzoites) enriched for the cell cycle phases of *Toxoplasma* endodyogeny. To visualize the cell cycle patterns, parasite clusters in each heat map were ordered by the progression of G1 to S/M/C peak transcript expression. Bradyzoite clusters (BAG1+) were included to the right (separated by dashed line) in each heat map. Note the G1 enriched profile of the bradyzoite clusters from astrocytes. [B.] The cell cycle heat map of in vivo bradyzoites compared to lab-adapted tachyzoites grown at pH8.2 or pH7.0 (reproduced from Fig. 2E) is included here to aid comparisons of G1-enhanced gene expression of alkaline-stressed tachyzoites to the G1-enhanced profile of brady-brady replicating clusters in [A.] above. **[C.]** Relative mRNA expression (GADPH1 normalized) of bradyzoite- and tachyzoite-specific mRNAs (left graph) and mRNAs encoding growth markers (right graph) in all BAG1+ clusters are presented (see Fig. 5B for further mRNA analysis details). GRA1 mRNA was included as a constitutive mRNA control. See Figure 5B for other gene details. Note the BAG1+ cluster (C8) from Day-5 HFF cells showed the lowest bradyzoite-specific mRNA expression.

Figure S5. Supplemental results for Figures 6 and 7.

[A.] CBA/j mice were inoculated i.p. with 10,000 bradyzoites, or 10,000 FTz- or STz-parasites from HFF cell cultures. At day 14 and 30 PI the indicated tissues were harvested, total DNA was extracted, and parasite

burden determined following amplification of the B1 gene compared against a standard curve. Significance (****, p<0.0001; ***, p<0.001; *, p<0.05) is indicated. **[B.]** Relative parasite burden in three organs is compared. In brain at 14 d.p.i., bradyzoite vs FTz- or STz-parasites are significantly different from each other (p<0.01), while at 30 d.p.i. only bradyzoite vs FTz-parasites and STz-parasite infected mice showed significantly different parasite burdens (p<0.05). **[C.]** Flow cytometry layouts of PECS taken at 5 d.p.i. (top) and 9 d.p.i. (bottom) from mice infected with parasites from above (see Fig. 7D quantification). Numbers in red = mean ± SD for intracellular parasites (top) and extracellular parasites (bottom) (n=5/group). **[D.]** Quantification of B1 gene analysis in PECS, brain, and lung at 5 and 9 d.p.i. from infected mice. Significance for all results were determined via One-way ANOVA with multiple comparisons (* p-value < 0.05, ** p<0.01, *** p<0.001, **** p<0.0001). Note that FTz-parasite infections were detected early in lung tissue.

Database S1. Total RNA-sequencing.

Work sheet definitions. Gene expression lists for in vivo bradyzoites (40 d.p.i) and in vitro tachyzoites grown in normal (36 h) or pH8.2 media (48 h). mRNA ranking and percentile values are included for in vivo bradyzoites and in vitro tachyzoites are included in separate work sheets. Bradyzoite and tachyzoite mRNA expression for uncharacterized genes are compared. The top expressed mRNAs (i.e. composition of the upper 50% of mRNAs) for in vivo bradyzoites and in vitro tachyzoites are compared. Finally, lists for selected G1 and S/M genes used to construct a cell cycle heat map are included.

Database S2 single cell RNA-sequencing.

Work sheet definitions. Gene expression lists for scRNA-sequencing of six samples are included in separate sheets; Day-2, -5, and -7 infected astrocytes or HFF cells. Selected scRNA-seq data of Day-7 parasites from astrocytes is included as an example of the mRNA series used to construction the cell cycle heat maps.
